# The Use of Machine Translation for Outreach and Health Communication in Epidemiology and Public Health: Scoping Review

**DOI:** 10.2196/50814

**Published:** 2023-11-20

**Authors:** Paula Sofia Herrera-Espejel, Stefan Rach

**Affiliations:** 1 Department Epidemiological Methods and Etiological Research Leibniz Institute for Prevention Research and Epidemiology - BIPS Bremen Germany; 2 Leibniz ScienceCampus Digital Public Health Bremen Germany

**Keywords:** machine translation, public health, epidemiology, population-based, recruitment, outreach, multilingual, culturally and linguistically diverse communities

## Abstract

**Background:**

Culturally and linguistically diverse groups are often underrepresented in population-based research and surveillance efforts, leading to biased study results and limited generalizability. These groups, often termed “hard-to-reach,” commonly encounter language barriers in the public health (PH) outreach material and information campaigns, reducing their involvement with the information. As a result, these groups are challenged by 2 effects: the medical and health knowledge is less tailored to their needs, and at the same time, it is less accessible for to them. Modern machine translation (MT) tools might offer a cost-effective solution to PH material language accessibility problems.

**Objective:**

This scoping review aims to systematically investigate current use cases of MT specific to the fields of PH and epidemiology, with a particular interest in its use for population-based recruitment methods.

**Methods:**

PubMed, PubMed Central, Scopus, ACM Digital Library, and IEEE Xplore were searched to identify articles reporting on the use of MT in PH and epidemiological research for this PRISMA-ScR (Preferred Reporting Items for Systematic Reviews and Meta-Analyses extension for Scoping Reviews)–compliant scoping review. Information on communication scenarios, study designs and the principal findings of each article were mapped according to a settings approach, the *World Health Organization monitoring and evaluation* framework and the *service readiness level* framework, respectively.

**Results:**

Of the 7186 articles identified, 46 (0.64%) were included in this review, with the earliest study dating from 2009. Most of the studies (17/46, 37%) discussed the application of MT to existing PH materials, limited to one-way communication between PH officials and addressed audiences. No specific article investigated the use of MT for recruiting linguistically diverse participants to population-based studies. Regarding study designs, nearly three-quarters (34/46, 74%) of the articles provided technical assessments of MT from 1 language (mainly English) to a few others (eg, Spanish, Chinese, or French). Only a few (12/46, 26%) explored end-user attitudes (mainly of PH employees), whereas none examined the legal or ethical implications of using MT. The experiments primarily involved PH experts with language proficiencies. Overall, more than half (38/70, 54% statements) of the summarizing results presented mixed and inconclusive views on the technical readiness of MT for PH information.

**Conclusions:**

Using MT in epidemiology and PH can enhance outreach to linguistically diverse populations. The translation quality of current commercial MT solutions (eg, Google Translate and DeepL Translator) is sufficient if postediting is a mandatory step in the translation workflow. Postediting of legally or ethically sensitive material requires staff with adequate content knowledge in addition to sufficient language skills. Unsupervised MT is generally not recommended. Research on whether machine-translated texts are received differently by addressees is lacking, as well as research on MT in communication scenarios that warrant a response from the addressees.

## Introduction

### Background

Public health (PH) and epidemiology are increasingly challenged by decreasing response proportions, in general, and an underrepresentation of culturally and linguistically diverse (CALD) communities, in particular [1]. Such underrepresentation increases the risk of biased estimates and, therefore, might limit the generalizability of findings in population-based research [2-7]. Ultimately, it might hinder the inclusion and involvement of these communities in disease prevention, surveillance efforts, and emergency response. In PH outreach and information campaigns, reaching CALD populations often poses greater difficulties than reaching other groups. As a result, these groups are challenged by 2 effects: the medical and health knowledge is less tailored to their needs, and at the same time, it is less accessible to them. These effects will only increase in importance as migration owing to globalization, global conflict, and economic inequalities increasingly shapes our societies toward multiculturality.

Using personalized recruitment material is an effective approach to engage individuals from CALD groups in population-based studies [8]. The choice of language matters because language barriers often result in their disengagement with PH initiatives [9-11]. If recipients are not able to comprehend transmitted information in the first place, they cannot react to it or provide an informed response [12]. Inclusive outreach approaches in PH study material, such as simplifying technical language or using multilingual cover letters, have been proven to improve access to information, foster meaningful participation, and reduce study nonresponse [13-15].

PH officials and researchers often struggle to effectively reach and engage all target audiences evenly. Although sufficient knowledge about the cultural composition of the target populations may be available (ie, the necessity to use particular languages), budget limitations usually restrict how many professional translations can be prepared and used for PH communication and outreach efforts to start with. A further complication with printed outreach material is that the number of different language versions that can be sent out in a single letter is physically limited, but the preferred language of an individual often is not known; therefore, it is difficult to conduct targeted outreach with specific language versions tailored to each recipient.

The use of machine translation (MT) technology poses a potential solution to overcome language hurdles in multilingual populations and improve effective material dissemination. As a computerized system, MT is able to automatically translate text or speech from 1 source language to multiple output languages [16]. In clinical settings, the technology has already been used to lower language barriers and facilitate services independently of the spoken language of the physician [17]. In the context of PH and epidemiology, MT could also be used to increase outreach by providing cross-lingual access to information and supporting PH staff to optimize material translation workflows.

### Prior Work

To our knowledge, there are 4 recent systematic reviews that cover aspects of the use of translation technologies in medical and clinical settings. In 2018, Dew et al [18] published a review on how the development of MT technology could be useful to assist one-way communication among individual stakeholders. In 2020, Frampton et al [19] systematically mapped digital tools for the recruitment and retention of participants in randomized controlled trials. Although the authors did not specifically address MT or similar language technologies, one of their main takeaways was that few studies address its use to support underserved groups. A year later, Thonon et al [20] published a review on the use of mobile apps to facilitate dialogue between health care professionals and CALD individuals with low language proficiency levels. In 2022, Vieira [21] published a review with a focus on the use of MT in medical and legal settings as 2 separate cases of translations of highly specialized vocabulary. The paragraphs devoted to medical settings mostly focused on one-to-one communication examples, mainly corroborating the findings of Dew et al [18].

In addition to these systematic reviews, other studies have assessed the use of MT in different health settings. Panayiotou et al [22] provided a methodical evaluation of 15 Apple iPad-compatible language translation apps to facilitate conversations between health care providers and patients in Australia; aside from its geographically bounded context, the study centers on native mobile apps for one-to-one communication. Nurminen and Koponen [23] outlined several applications of MT for increasing information accessibility in humanitarian settings (eg, an armed conflict, a natural disaster, or an epidemic), including a paragraph devoted to discussing community-based health, as well as safety and security information. Although relevant to PH, the overview neither specifically reviews other contexts nor identifies patterns in the literature regarding the state of readiness of MT for PH settings.

These earlier publications are mostly confined to reporting literature on the use of MT for real-time bilingual person-to-person communication. The technology is mainly studied as an on-premise solution to support medical service provision in spoken interactions between specific groups of patients (eg, tourists, refugees, or expatriates) and health care staff (eg, general practitioners, caregivers, or paramedics) [24-28]. Only a few of the articles explore the use of multilingual translation tools for disseminating PH information to specific target audiences [29] or for population-wide health initiatives [30].

### The Goal of This Study

The objective of this scoping review was to systematically map the use of MT for conducting PH outreach, with a particular focus on population-based recruitment methods. As a first step, we identify the information exchange scenarios in which MT technology is used to facilitate essential PH operations in different health and care settings. Second, we provide an overview of the types of study designs and research instruments for monitoring and evaluating the use of MT in these cases. Third and last, we synthesize the reported findings, benefits, and risks in relation to technical, socioeconomic, and ethicolegal technology readiness levels.

## Methods

### Search Strategy and Selection Criteria

This scoping review was preregistered on the Open Science Framework on February 11, 2022 [31], and conducted in accordance with the updated guidance on scoping reviews of the JBI Manual for Evidence Synthesis [32] as well as the PRISMA-ScR (Preferred Reporting Items for Systematic Reviews and Meta-Analyses extension for Scoping Reviews) checklist (Multimedia Appendix 1 [33]) [34,35].

This scoping review exclusively includes peer-reviewed original research describing and assessing the use and suitability of MT for written texts for the purpose of improving collective outreach as well as the response and involvement of participants in the fields of epidemiology and PH, regardless of the specific target interventions or health areas involved. Given the technical nature of the research topic, peer-reviewed conference papers were also included. In addition, articles reporting guidelines or consensus statements concerning the use of MT in PH settings were included. The scoping review considers only studies written in English and published from 2007 onward, a year after the launch of the first fully web-based MT system and the publication of the first reference framework for MT quality assurance (the EN15038 standard) [36]. Studies of individual care or counseling settings (eg, practitioner and patient) were excluded because, in these settings, MT is used for spoken two-way communication. Textbox 1 presents the eligibility criteria.

**Eligibility criteria for the scoping review.**

**Inclusion criteria**

- Article type
  Peer-reviewed original research
  Peer-reviewed conference papers
  Consensus statements concerning the use of machine translation in public health settings
- Language
  Studies published in English
- Time span
  Studies published after January 2007
- Study design
  Empirical studies

**Exclusion criteria**

- Article type
  Non–peer-reviewed research or gray literature
- Language
  Studies not published in English
- Time span
  Studies published before 2007
- Study design
  Nonempirical studies
  Studies of individual care or counseling settings

### Search Strategy

Searches were conducted in PubMed (MEDLINE), PubMed Central, Scopus, ACM Digital Library, and IEEE Xplore.

As recommended by JBI, the search string was constructed according to the population or participants, concept, and context (PPC) framework [32]. No specific restrictions were used to define the study populations. The concept was defined by terms related to automatic translation technology and the context by defining settings for population-based communication in PH, epidemiology, and community-based health care (Multimedia Appendix 2).

Initially, the search string was created to query the PubMed search engine and thereafter adapted to PubMed Central, Scopus, IEEE Xplore, and ACM Digital Library (the search terms are listed in Multimedia Appendix 2). Where available, database-specific index terms were added (eg, Medical Subject Headings [MeSH] terms for PubMed). The search was restricted to abstracts and titles. The search strategy was refined with the assistance of a professional librarian. All searches were executed on January 31, 2022, and updated on March 3, 2023.

After deduplication and the application of the exclusion criteria (ie, language not English, publication before 2007, and non–peer-reviewed articles), both authors (PSH-E and SR) independently screened the titles and abstracts of all remaining records using the R packages *revtools* [37] and *metagear* [38], which provide tools for semiautomatic deduplication and title or abstract screening. Disagreements were discussed and resolved by reaching a consensus. If necessary, full texts were consulted.

### Data Extraction, Synthesis, and Analysis

Data extraction was conducted using a standardized data extraction template to extract bibliographic characteristics, health information exchange scenarios, research objectives and corresponding study designs, and technical characteristics of the MT tools used, as well as to identify the principal findings in the selected articles.

Health information exchange scenarios were assessed using a settings approach to health promotion [39]. We extracted and classified data regarding the (1) transmitters and recipients of translated materials, (2) types of translated materials, (3) types of MT systems and the source and target languages studied, and (4) nature of the use of MT in PH procedures as unsupervised (ie, without editing efforts) or supervised (ie, combined with editing efforts).

Research objectives were assessed according to the World Health Organization (WHO) monitoring and evaluation (M&E) framework [40], which is useful to map the research and development of digital health technologies according to their stage in the innovation maturity life cycle. We then classified the articles as either monitoring studies or evaluation studies. We considered monitoring studies to be those involving research on the technical quality and stability of MT (eg, technology assessments and comparative experiments) and evaluation studies to be those reporting on the appraisals of the technology-based interventions over time (eg, usability, affordability, and economic cost-effectiveness studies), as well as implementation research for integrating developed systems within broader PH workflows.

To assess the principal findings, we extracted sentences reporting quantitative and qualitative outcomes from the results sections. Following the *service readiness level* framework of evidence proposed by Hughes et al [41], we organized the statements as concerning technical, socioeconomic, or ethicolegal readiness levels of MT technology. On the basis of a manual sentiment analysis, we then detected the tonality of each text and classified them as positive, negative, or neutral.

## Results

### Search Outcomes

**Figure 1:**
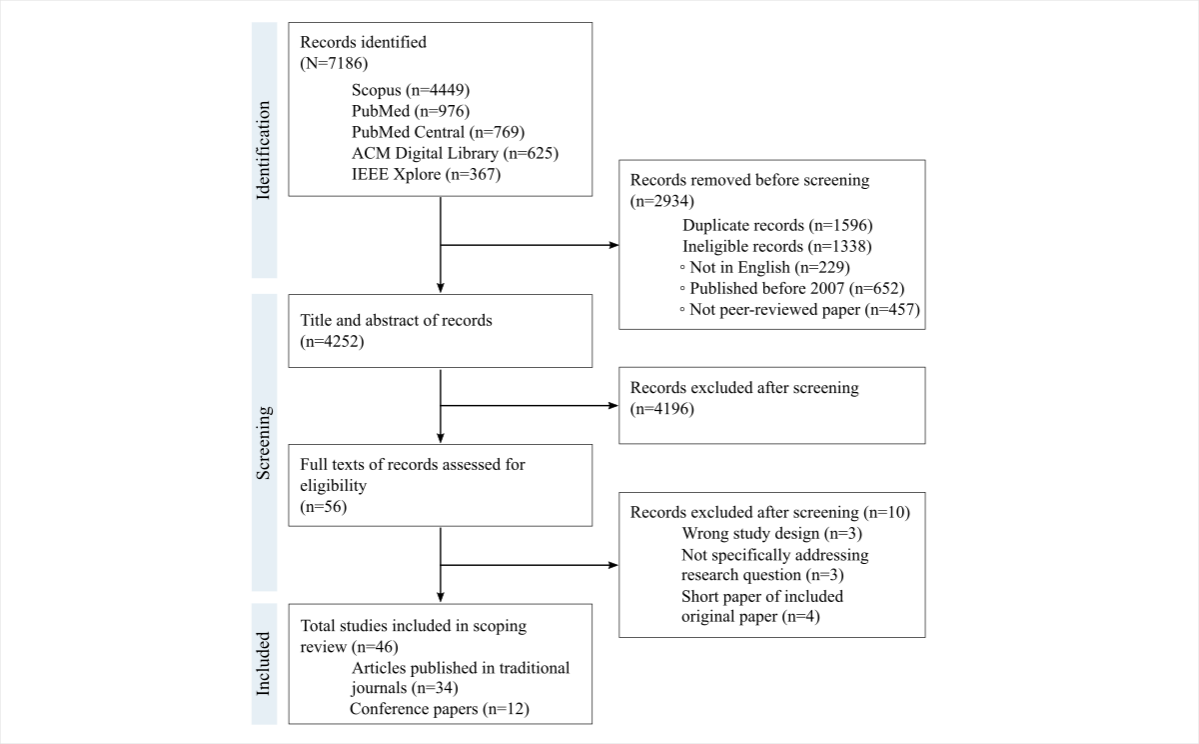
Flow diagram of the search and study selection process following the PRISMA-ScR (Preferred Reporting Items for Systematic Reviews and Meta-Analyses extension for Scoping Reviews) guidelines.

Conducted on January 31, 2022, and updated on March 3, 2023, the search yielded a total of 7186 records, of which 2934 (40.83%) were removed (1596/2934, 54.4% duplicates and 1338/2934, 45.6% not meeting the eligibility criteria). A review of the titles and abstracts of the remaining 4252 records resulted in 56 (1.32%) being selected for a full-text screening. From these 56 articles, 10 (18%) were removed for not meeting the study design criteria, not specifically addressing the research question, or for providing duplicate information from another included paper (Multimedia Appendix 3), and 46 (82%) were included in the systematic scoping review (Figure 1; Multimedia Appendix 4 [29,42-86]).

The records were published between 2009 and 2023 as either conference papers (12/46, 26%) [48,53,55,60,61,63,65,​^71^,^78^,^80^,^82^,^84^] or articles in traditional journals (34/46, 74%) [29,42-47,49-52,54,56-59,62,64,66-70,72-77,79,81,83,85,86] (Multimedia Appendix 4).

### PH Information Exchange Scenarios

Four types of information transmitters (ie, the end users of MT) could be identified: PH departments and research institutions (21/46, 46%) [29,49,50,52-54,56,59,60,62,63,65,70,73-78,​^80^,^86^]; clinical and hospital staff (15/46, 33%) [44-47,51,58,61,66,68,69,71,72,79,81,85]; international and national health organizations, such as the WHO, the US Centers for Disease Control and Prevention, and the UK National Health Service (8/46, 17%) [42,43,55,57,64,82-84]; and developers of web-based health information platforms (eg, Cochrane) or social media outlets (eg, Facebook; 2/46, 4%) [48,67].

The types of PH materials translated with MT fell into 6 broad categories: official guidelines and educational resources (11/46, 24%) [42,43,53,62,67,70,75,79,80,82,83], simplified medical information and lexica (11/46, 24%) [48,54,55,61,63,68,69,71,81,85,86], PH promotional material (10/46, 22%) [29,49,50,59,60,64,65,76-78], instruction handouts (6/46, 13%) [51,52,57,58,66,72], academic research (6/46, 13%) [44-47,73,84], and survey instruments (2/46, 4%) [56,74].

The information receivers (ie, the end users of translated material) could be categorized into 5 types: the wider population as targets of PH material offline (18/46, 39%) [29,48,51,53,55,58,59,62,64,65,67,68,74,76-78,80,86] or on the world wide web (7/46, 15%) [42,43,49,70,75,79,82], patient groups or communities (10/46, 22%) [50,52,57,61,​^63^,^66^,^69^,^72^,^81^,^85^], clinical and hospital staff (7/46, 15%) [44-47,54,71,83], and PH professionals (4/46, 9%) [56,60,73,84].

**Figure 2:**
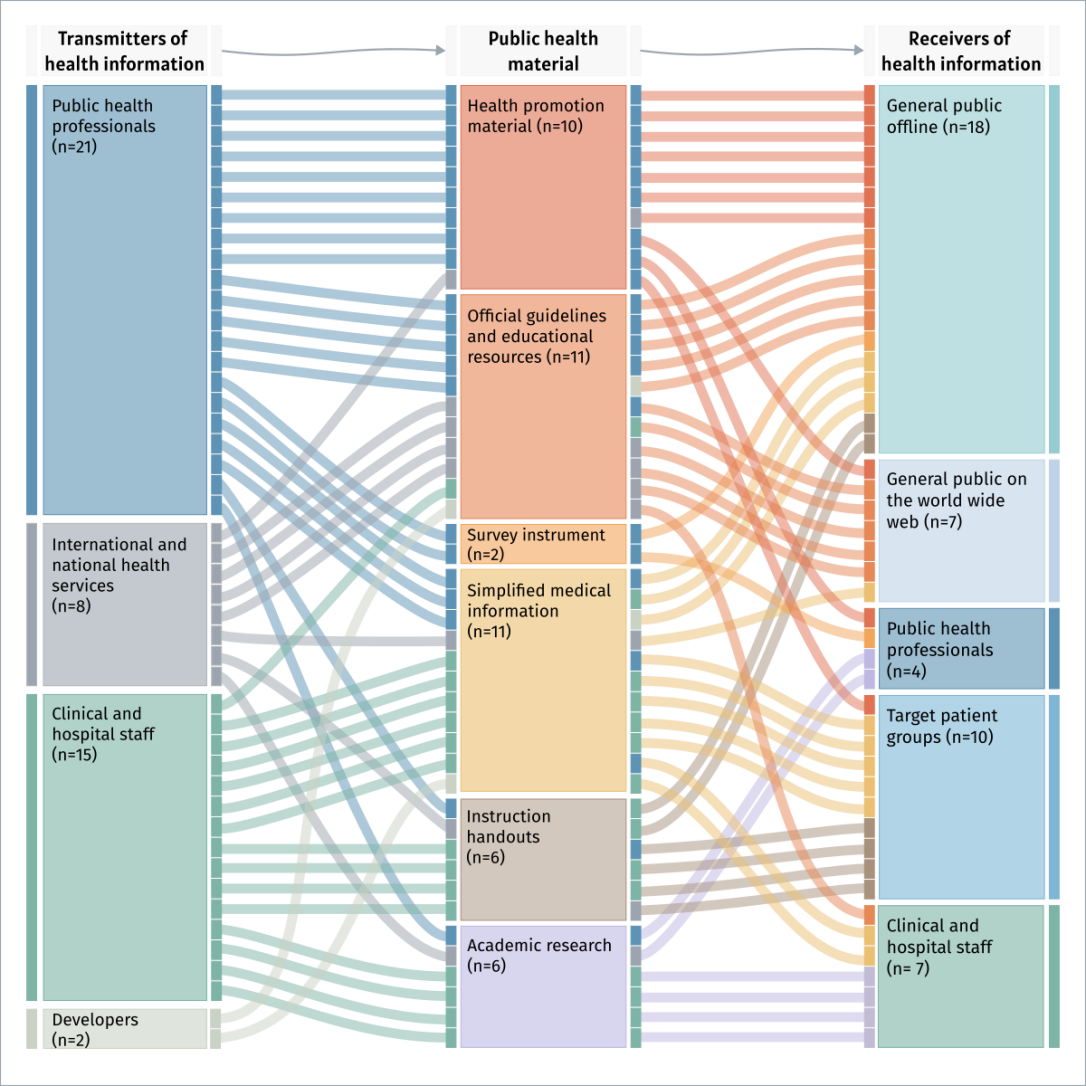
Public health information exchange scenarios: transmitters and receivers of public health information and the types of public health materials.

Figure 2 provides a Sankey diagram visualizing PH information exchanges supported with the use of MT technology between groups of transmitters and receivers across the selected articles (Multimedia Appendix 5 [29,42-86]).

Overall, the most frequent use case of MT for collective communication in a PH setting is between PH staff and the public receiving paper-based health information (ie, offline; 14/46, 30%) [29,53,56,59,60,62,65,73,74,76-78,80,86] such as health promotion material (6/14, 43%) [29,59,65,76-78] or educational resources (3/14, 21%) [53,62,80]. The second most frequent use cases were exchanges involving clinical and hospital staff (10/46, 22%) [51,58,61,66,68,69,72,79,81,85] such as nurses or emergency wards disseminating simplified medical information (5/10, 50%) [61,68,69,81,85] or preparing instruction handouts for targeted audiences (4/10, 40%) [51,58,66,72].

Most of the articles (40/46, 87%) [29,42-48,50-63,66-69,71-74,77,79-86] specified the type of procedure for the use of MT as either unsupervised (30/40, 75%) [42-48,50-52,54-58,63,67-69,71-73,79-85] or supervised (10/39, 26%) [29,53,59-62,66,74,77,86] (Multimedia Appendix 5). Unsupervised MT was used for enabling translation plug-ins on PH websites (9/30, 30%) [42,43,48,67,69,79,81-83], translating PH material via web-based MT services (8/30, 27%) [50-52,56-58,68,72], and translating English content into the researchers’ language (7/30, 23%) [44-47,73,80,84], as well as to investigate the risks of mistranslation and translation quality (5/30, 17%) [54,55,63,71,85]. Supervised MT procedures included postediting (6/10, 60%) [29,53,59,60,77,86] or pre-editing of source language (2/10, 20%) [61,62], as well as back translations (2/10, 20%) [66,74] of sample texts on the web and paper-based material.

The tested MT software tools were either freely available on the web from commercial technology vendors or were in-house built systems created by the research teams themselves (Multimedia Appendix 5). Regarding commercial vendors, Google Translate was the most used translation engine (28/46, 61%) [29,43-45,48,50-52,54-59,62,63,66,69,72,74,76-83,86], followed by Microsoft Bing (5/46, 11%) [44,47,61,69,79] and DeepL Translator (2/46, 4%) [73,86], among others. All these systems were used as domain-agnostic systems and not pretrained on specific language corpora. All articles regarding in-house built systems (9/46, 20%) [61,63,69,71,76,79,80,82,84] presented a prototype demonstration of domain-specific MT systems specifically trained on PH-related and medical vocabulary. The studies comparing these systems against each other (4/9, 44%) [71,79,80,84] advocate for using in-house built systems for shorter text with medical terminologies in long-term projects, whereas off-the-shelf systems may be used for more general information. In relation to each other, the evidence does not clearly favor 1 translation engine over another. Instead, it suggests that the choice among systems depends on the language pairs and the vocabulary domain used in the material. Provided that the texts are not exclusively reliant on specific terminologies, domain-agnostic solutions are equally suited for handling short-text translations.

Of the 46 articles, 40 (87%) [29,42-48,50-63,​^66^-^69^,^71^-^74^,^77^,^79^-^86^] studied the use of MT to translate from 1 source language into 1 or several target languages (Multimedia Appendix 5). Of these 40 articles, 37 (93%) [29,42-45,48,50-63,66-69,71-74,77,79-85] specified the source language, whereas 35 (88%) [29,42-45,48,50-52,54-63,​^66^,^67^,^69^,^71^-^74^,^77^,^79^-^86^] specified the target language. Of the 12 source languages, English was the most commonly evaluated (32/37, 86%) [29,42-45,48,50-60,62,66-68,​^71^,^72^,^74^,^77^,^79^-^85^], followed by French (4/37, 11%) [48,69,79,80,84], German (3/37, 8%) [69,79,80], and Spanish (3/37, 8%) [79,80,84]. MT was tested in translating texts from English into at least 44 other languages, with Spanish (17/35, 49%) [29,48,50-52,​^57^-^60^,^66^,^72^,^74^,^79^,^80^,^83^-^85^], Chinese (13/35, 37%) [50-52,56,58,62,72,74,77,81-83,85], and French (7/35, 20%) [52,71,74,79,80,83,84] being the most frequent target languages. Within this subset of 35 articles, 25 (71%) [29,42-45,50-52,54,56,57,59-63,66,69,72-74,77,79,80,85] conducted studies in specific geographies targeting populations and communities with limited language proficiency. Of these 25 articles, 19 (76%) [29,50-52,56,57,59-63,66,69,​^72^,^74^,^77^,^79^,^80^,^85^] targeted individuals with limited English proficiency, mainly residing in the United States (17/19, 89%) [29,50-52,56,57,59-61,63,66,69,72,77,79,80,85].

### Study Designs According to the WHO M&E Framework

In accordance with the WHO M&E framework [40], we identified 6 types of research designs across the selected articles (Table 1; Multimedia Appendix 6 [29,42-86]): MT technology assessments (study type 1; 23/46, 50%) [29,43-46,48,​^50^-^52^,^54^-^59^,^62^,^68^,^72^-^74^,^77^,^85^,^86^], technology stability standards (study type 2; 3/46, 7%) [66,71,81], prototype demonstrations (study type 3; 8/46, 17%) [61,63,69,79,80,82-84], usability studies (study type 4; 4/46, 9%) [42,47,64,65], economic evaluations (study type 5; 4/46, 9%) [49,70,75,78], and implementation research (study type 6; 4/46, 9%) [53,60,67,76]. Nearly three-quarters (34/46, 74%) of the articles [43-46,48,50-52,54-59,61-63,66,68,69,71-74,​^76^,^79^-^86^] conducted monitoring studies (ie, study types 1, 2, and 3), whereas more than a quarter (12/46, 26%) [42,47,49,53,60,64,65,67,70,75,76,78] conducted evaluation studies (ie, study types 4, 5, and 6).

**Table 1:** Categorization of studies according to the World Health Organization monitoring and evaluation framework (n=46).

Study type and research design			Studies, n (%)
**Monitoring studies: functionality and stability of MT^a^ at predefined levels of quality**			
	1. MT technology assessments: studies assessing MT quality, functionality, and performance	23 (50)	
	2. Technology stability standards: studies proposing standards or criteria for MT quality assurance	3 (7)	
	3. Prototype demonstrations: studies reporting on the development and design of an in-house built MT-based system	8 (17)	
**Evaluation studies: MT technology in health-related settings**			
	4. Usability studies: studies addressing end-user attitudes, perceptions, and responses when using the prototype system and assessing how easily end users can interact with the system	4 (9)	
	5. Economic evaluations: studies addressing accessibility, availability, or affordability of the system	4 (9)	
	6. Implementation research: studies around the implementation of MT technology within a broader (public) health system architecture	4 (9)	

^a^MT: machine translation.

The monitoring studies adopted standard MT evaluation methods to measure the quality of MT output across various samples of health information material. Most of these studies focused on studying MT quality in terms of structural accuracy (28/34, 82%) [29,43,45,46,48,50-52,54-58,61,63,68,69,72-74,77,79-84,​^86^] and fluency in unsupervised MT procedures (17/34, 50%) [44-46,50,51,54,56,57,59,62,66,71,72,77,80,81,85]. A quarter (8/28, 29%) of the articles [61,62,69,71,79,80,82,84] assessing structural accuracy supplemented their findings with standard automatic evaluation methods to verify the quality of MT output in comparison with the output of professional human translators. Flesch-Kincaid grade level scores and content analysis techniques were used to measure the readability levels and meaning preservation of the translated sentences. In a few of the articles (6/34, 18%) [50,51,57,58,66,72], MT was also evaluated in terms of the risk severity of mistranslation (ie, the degree of negative impact on the patient’s health outcome because of a wrong translation). Studies investigating postediting (4/34, 12%) [29,59,77,86] or back translation (2/34, 6%) [66,74] focused on identifying error patterns or measuring the amount of time saved, whereas pre-editing (2/34, 6%) [61,62] was investigated to understand the ability of MT to handle PH jargon and medical terminologies.

In their experiments, some of the studies (4/34, 12%) [29,43,59,81] extracted sentences from global PH (2/4, 50%) [43,81] and local PH promotion documents (2/4, 50%) [29,59]. Others (7/34, 21%) [48,51,58,61,66,68,72] carried out their experiments with general patient care instructions (2/7, 29%) [66,68], with side effects lists and directions for the use of prescribed drugs (3/7, 43%) [48,51,61], and from free-text or commonly used sentences in discharge instructions (2/7, 29%) [58,72]. A few of the articles (5/34, 15%) [44-47,54] used sentences from nursing abstracts (4/5, 80%) [44-47] and technical glossaries and dictionaries (1/5, 20%) [54]. Most of these experiments (27/34, 79%) [29,43-46,48,50-52,​^54^-^59^,^61^,^63^,^66^,^68^,^74^,^77^,^80^-^82^,^85^,^86^] recruited participants among PH professionals and certified translators with high proficiency in target and source languages or some experience with PH vocabulary. In general, discussions on ethical issues and quality inefficiencies across different languages did not address the impact of MT on possible information divides.

The evaluation studies deployed qualitative research instruments to understand how different types of end users view the adoption of MT technology in their information communication processes. Along with semistructured interviews, cognitive workflow analyses were used to understand current practices and the actual use of MT for multilingual document production workflows by PH departments. In addition, many of the studies (8/12, 67%) [42,47,49,53,64,65,67,78] used structured questionnaires and semistructured interviews to assess the perceived usefulness of MT (4/8, 50%) [42,47,64,67] and attitudes toward its adoption (4/8, 50%) [49,53,65,78] in such cases. Some of the articles (3/12, 25%) [53,60,76] sought to determine the practicality of implementing MT in combination with postediting efforts into local PH department workflows by timing and measuring the translation error rate of different translation procedures. Of the 12 studies, 4 (33%) [47,60,65,78] interviewed PH personnel and experts, and 2 (17%) [49,70] analyzed the availability of languages in PH web pages. Only 1 (8%) [42] of the 12 studies surveyed individuals in a real-world setting (ie, Facebook posts) to understand intelligibility or comprehension problems produced by MT in daily life situations on the web.

### Technical, Socioeconomic, and Ethicolegal Readiness Levels

**Figure 3:**
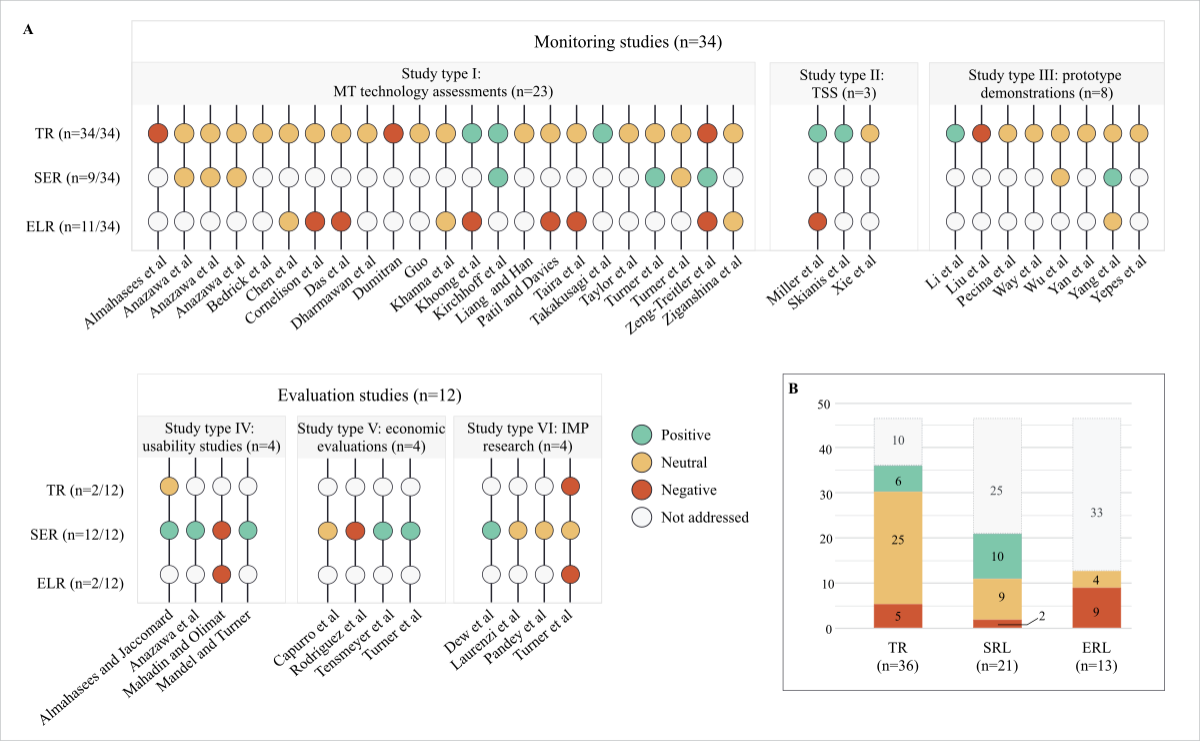
Appraisal of study results. (A) Positive, negative, and mixed findings on the use of machine translation (MT) in public health settings by type of study and technology readiness dimensions. (B) Aggregate of final statements (N=70) by technological readiness levels across the 46 selected articles. IMP: implementation research; ELR: ethicolegal readiness; SER: socioeconomic readiness; TR: technical readiness; TSS: technology stability standards.

A total of 70 statements were identified as principal findings in the discussion and conclusions sections within the 46 articles (Multimedia Appendix 7 [29,42-86]). The majority (36/46, 78%) of the articles under review [29,42-46,48,​^50^-^52^,^54^-^59^,^61^-^63^,^66^,^68^,^69^,^71^-^74^,^76^,^77^,^79^-^84^,^86^] drew conclusions regarding the level of technical readiness of MT, nearly half (21/46, 46%) [29,42,44-47,49,53,59,60,​^64^,^65^,^67^,^70^,^75^-^78^,^80^,^83^,^85^] considered MT’s socioeconomic readiness, and more than a quarter (13/46, 28%) [50-52,57,58,64,66,68,72,76,83,85,86] discussed the ethicolegal readiness of the translation outcomes for PH operations. Overall, one-fifth (16/70, 23%) of the statements within the articles expressed optimism about the use of MT for PH purposes [29,42,47,53,58,59,61,65,66,71,73,75,78,83,85], whereas another one-fifth (16/70, 23%) was pessimistic [43,51,52,55,58,63,64,66,68,70,72,76,85], and the remainder (38/70, 54%) presented mixed or inconclusive results [29,42,44-52,54,56,57,60,62,67-69,72,74,76,77,79-84,86]. Optimistic, pessimistic, and neutral statements are accounted for by green, red, and yellow circles, respectively, in Figure 3 [29,42-86].

Concerning final statements on the technical readiness of MT, three-quarters (25/36, 69%) [29,42,44-46,48,50-52,54,56,​^57^,^62^,^68^,^69^,^72^,^74^,^77^,^79^-^84^,^86^] of the articles were inconclusive and expressed cautionary optimism for translating PH material. Some of these articles (5/25, 20%) [29,62,74,77,86] recommended using a combination of MT and postediting efforts to improve translation quality and productivity. Of the 25 articles, 2 (8%) [61,62] also recommended using pre-editing efforts, such as controlled language and vocabularies, to reduce the need for postediting efforts. Articles addressing the socioeconomic readiness of automatic translations (21/46, 46%) [29,42,44-47,49,53,59,60,64,65,67,70,75-78,80,83,85] concluded either with optimistic (10/21, 48%) [29,42,47,53,59,65,75,78,83,85] or mixed results (9/21, 43%) [44-46,49,60,67,76,77,80], whereas a couple presented pessimistic results (2/21, 10%) [64,70]. On the one hand, these articles confirmed the enthusiasm of PH workers to adopt MT to increase cost-effectiveness as well as provide diverse material to wider audiences. On the other hand, they also stressed the importance of preparing the workforce to use the technology and ensuring that standard processes are created in light of PH equity goals. Regarding ethical and legal readiness, none of the articles mentioning the topics (n=13) concluded with optimistic statements; the findings were mostly negative (9/13, 69%) [51,52,58,64,66,68,72,76,85] and a few were mixed (4/13, 31%) [50,57,83,86]. In general, the articles stressed that the technology represents noteworthy communication risks, namely owing to a varying translation accuracy across languages. A few studies (4/13, 31%) [52,66,72,86] also pointed out that the commercial vendors’ algorithms are not verifiable by the researchers or staff, resulting in a loss of control when not combined with editing efforts.

## Discussion

### Principal Findings

In our scoping review, we sought to systematically identify and map existing peer-reviewed literature on the use of MT for population-based outreach, with a particular interest in its use for recruiting participants for PH and epidemiological research. None of the included articles (n=46), published between 2009 and 2023, tested MT for recruiting participants to population-based studies or in scenarios where a response from addressees is expected. Research on the use of MT for PH activities is still in its early stages, primarily concentrating on assessing the technical readiness for one-way written communication between PH officials and addressed audiences. The majority of information transmitters (ie, the end users of MT) were PH professionals in PH departments and research, clinical and hospital staff, or staff at international and national health organizations. PH materials translated with MT were predominantly official guidelines and educational resources, simplified medical information, or PH promotional material. The intended target audiences (ie, the receivers of translated material) were the wider population (both offline and seeking information on the world wide web), patient groups, or professionals in PH and clinical settings. Nearly three-quarters (34/46, 74%) of the articles reported monitoring studies, with the remaining quarter (12/46, 26%) reporting evaluation studies.

### Research on the Use of MT for PH Activities Is Still Nascent

The current focus of research is mostly concentrated on understanding the extent to which machine-translated output is reliable and stable enough for translating specific sample texts, while placing less emphasis on the feasibility of its use in real-world settings. Published study types mostly provided technical maturity assessments of MT (eg, in exploratory research, experimental proofs of concept, and implementation research studies).

The majority of the studies (28/46, 61%) [29,43,45,46,48,50-52,54-58,61,63,68,69,72-74,77,79-84,86] solely focused on MT accuracy errors and how to drive error rates down. Most of the articles (23/46, 50%) [29,43-46,48,50-52,54-59,62,68,72-74,77,85,86] provided technical assessments, and in most of the cases (14/23, 61%) [29,43-46,48,54-56,59,62,73,74,77], they studied neither the reliability of the technology for specific target audiences nor the potential risks of mistranslation. Although articles often specified the type of MT algorithm as either statistical MT or neural MT (Multimedia Appendix 5), none systematically compared the algorithms or reported on specific advantages or disadvantages. Therefore, it is not clear whether the type of algorithm has any relevance for using MT in PH scenarios.

A handful of studies (9/46, 20%) [61,63,69,71,76,79,80,82,84] reported ongoing research in the development of in-house software, pretrained on specific vocabulary. These systems were reported to outperform off-the-shelf models (eg, Google Translate and DeepL Translator), namely when translating shorter text with specialized terminologies, such as those used in medical guidelines or prescriptions. The fact that the technology is evolving and can now be trained in PH and biomedical vocabulary sheds light on future possibilities to meet the needs of staff working with more complex PH material. However, the current state of evaluations on the advantages and disadvantages of the off-the-shelf systems over internally developed models does not yet allow PH researchers to model the best use of both systems during specific stages of material production. Provided that PH material does not heavily rely on domain-specific vocabulary, off-the-shelf MT solutions are sufficiently reliable in terms of translating shorter text. Given that these systems are predominantly free to use and easily adaptable to a translation workflow, proprietary models are relatively costly to develop and maintain, as well as scale to new vocabularies.

The literature tends to focus on evaluating the accuracy of supervised translations from the language of the working staff or researchers (typically English) to 1 or a few languages (in most cases, Spanish, Chinese, or French). The observed inclination to study English as a source can be attributed to the origin of the selected articles in this review. For most of the studies (19/46, 41%) [29,50-52,56,57,59-63,66,69,72,74,​^77^,^79^,^80^,^85^], the target audiences of interest were large linguistically diverse communities residing in predominantly English-speaking countries (eg, the United States, the United Kingdom, and Australia). Future studies could also aim to cover underrepresented languages beyond that of the largest linguistically diverse groups and continue exploring cases to support linguistically diverse PH staff. For now, a few of these studies (6/46, 13%) [29,59,66,74,77,86] tested MT in light of postediting efforts. As user-friendly MT applications become more accessible to the public and professionals, we can reasonably assume that the focus of MT research in PH might shift from generating texts with MT to generating texts that are optimized for MT, that is, the emphasis might shift from technical accuracy and postediting efforts to pre-editing of texts.

A limited number of articles (21/46, 46%) [29,42,44-47,49,53,59,60,64,65,67,70,75-78,80,83,85] investigated the societal acceptance of MT, mainly by surveying the attitudes of PH staff toward its adoption, formulating new concepts, and studying current practices and standards. The selected studies point to the conclusion that PH staff are enthusiastic and open to adopting MT in their workflows. Almost half (10/21, 48%) of the studies held positive attitudes toward the potential cost-effectiveness of using MT to increase public access to PH information. However, the technology has not been routinely adopted by PH departments owing to safety concerns, the loss of control over content, and the unquantified variability of the quality of translation between languages. There is a need to further identify relevant stakeholders for implementing and deploying MT, as well as to test proposed solutions in controlled environments with the end users of translated material.

Most of the experiments (31/46, 67%) were based on expert focus groups and surveying PH professionals, whereas only a few (3/46, 7%) explored end-user interactions, preferences, and perspectives in real-world settings. However, without real-world studies conducted outside laboratory settings and in field experiments, the user experience of the technology remains largely unknown. Only a few studies (8/46, 17%) [42,47,49,53,64,65,67,78] tested the usability and acceptability of MT in community settings. Future studies could explore, for example, end-user interactions with machine-translated text in daily life settings, while also continuing to survey PH professionals in digital environments and capturing their attitudes toward use and adoption, as well as measuring the actual information uptake by groups targeted with machine-translated materials compared with nontranslated materials alone.

Moreover, no article focused solely on the legal or ethical aspects of the use of MT for PH purposes. However, some of the studies (13/46, 28%) [50-52,57,58,64,66,68,72,76,83,85,86] did provide a generic consideration of ethical compliance aspects as part of their discussions. To the extent that these concerns were addressed, 2 (4%) of the 46 studies called attention to the fact that the commercial vendors’ algorithms are not transparent to researchers and staff. Investigating MT from an ethical perspective, such as its impact on the digital divide, and establishing standards for its adoption also remain pending in light of PH equity goals and the risk of harmful errors.

### No Current Research on MT in Two-Way Communication Scenarios

None of the reviewed studies specifically tested MT for the recruitment of participants in population-based research. The literature only covers the use of MT for communicating in PH settings that do not warrant a response from addressees. Most of the studies (27/46, 59%) [29,42,43,49-53,57-60,​^62^,^64^-^67^,^70^,^72^,^75^-^80^,^82^,^83^] focused on the use of MT for translating simple text in flyers, instructions, and general information sheets from 1 language into a selected few. Hardly any of the articles (44/46, 96%) [29,42-74,80-86] discussed cases where the technology was used to communicate with several linguistically diverse populations at once. Only 2 (4%) [75,79] of the 46 studies introduced the use of MT for emergency preparedness and outreach prompted by the COVID-19 pandemic. These cases remain examples of unidirectional communication between PH staff and addressed audiences who are not expected to provide a response in return.

One possible reason why MT has not been used for recruitment in population-based research may be that there is limited utility in providing translations of PH material into languages that are not spoken or read by researchers or field staff or in recruiting participants who cannot interact with the languages in which the study is offered. On the contrary, if studies are offered in multiple languages, they are usually prepared with research instruments and personnel pre-equipped with the skills to meet the language diversity of the study population. It is therefore rather unlikely that MT would be necessary for translating recruitment materials in the first place.

However, there are scenarios in which MT may prove beneficial in population-based recruitment; for example, in studies on children and adolescents, the actual study participants often speak the language of the country fluently, but their legal guardians, who have to consent to their children’s participation, might not be proficient in the language. Providing them with study information and consent forms in their preferred language might help them to understand what is asked from them and their children and, therefore, increase the probability that they will provide consent. However, for such purposes, ensuring a certain translation quality is crucial to meet ethical and legal requirements, but, as mentioned before, this review did not find much evidence of research regarding this problem.

Furthermore, providing multilingual invitations could also help PH employees to understand the demand for different languages at the population level. If addressees could be enabled to report their preferred languages back to PH staff, the collected data might be used to adapt ongoing or future studies to provide additional language support. Alternatively, addressees could be informed that participation is possible, contingent on being accompanied by a translator.

Finally, even if it is not possible to add each language preferred by potential study participants, using MT tools for PH study invitations would ensure that more addressees understand the content of the invitation letters, which, given their official appearance, might otherwise leave them uncertain regarding missing out on something important or even undermine trust in PH departments and reduce participation in future studies or initiatives.

### Limitations

Our findings should be considered with limitations. First, this review is limited to publications addressing the use of MT either as part of the research question or as a key point of discussion in the publications. It cannot be ruled out that MT might already be used as a routine tool, and therefore, its use is not reported in peer-reviewed papers. Second, we used an interpretative sentiment analysis to classify the principal findings for each article based on the extraction of selected statements. This exercise, although systematic and with the intention of objectivity, is prone to the authors’ interpretation of enthusiasm regarding the specific dimensions of digital technology maturity. Finally, the search was limited to articles published only in English, which might bias the results toward studies examining MT from or into English. There is also a possibility that articles published before 2007 could contain information relevant to the research question. However, because the technology has evolved exponentially in the last 2 decades, prior information is likely to be outdated and no longer applicable to current standards.

### Conclusions

Using MT in epidemiology and PH can enhance outreach to linguistically diverse populations. The translation quality of current off-the-shelf systems, such as Google Translate or DeepL Translator, is sufficient if postediting is a mandatory step in the translation workflow. Postediting of legally or ethically sensitive material requires staff with adequate content knowledge in addition to sufficient language skills. When preparing texts for translation, it is advisable to use shorter sentences and specifically mark domain-specific vocabulary for possible postediting. Unsupervised MT is generally not recommended. Research on whether machine-translated texts are received differently by addressees is lacking, as well as research on MT in communication scenarios that warrant a response from the addressees.

## Appendix

Multimedia Appendix 1 PRISMA-ScR (Preferred Reporting Items for Systematic Reviews and Meta-Analyses extension for Scoping Reviews) checklist.

Multimedia Appendix 2 Search terms for PubMed, PubMed Central, Scopus, IEEE Xplore, and ACM Digital Library.

Multimedia Appendix 3 List of studies excluded during full-text screening.

Multimedia Appendix 4 Study characteristics.

Multimedia Appendix 5 Transmitters, receivers, public health material, and machine translation engines.

Multimedia Appendix 6 Research study designs.

Multimedia Appendix 7 Authors’ sentiment on the principal findings in the discussion and conclusions sections.

## Abbreviations

CALD: culturally and linguistically diverse
M&E: monitoring and evaluation
MeSH: Medical Subject Headings
MT: machine translation
PH: public health
PPC: population or participants, concept, and context
PRISMA-ScR: Preferred Reporting Items for Systematic Reviews and Meta-Analyses extension for Scoping Reviews
WHO: World Health Organization

## Data Availability

The complete list of included studies and all data generated or analyzed during this study are included in this published article and the multimedia appendices.
