# Supplementary material for: The Use of Machine Translation for Outreach and Health Communication in Epidemiology and Public Health: Scoping Review
Source: JMIR Public Health Surveill. 2023 Nov 20;9:e50814. doi: 10.2196/50814 (PMC10696499; doi:10.2196/50814)
Supplement: Multimedia Appendix 2 [file publichealth_v9i1e50814_app2.pdf]

The use of machine translation for outreach and health communication in epidemiology and public health: scoping review

Paula S. Herrera-Espejel and Stefan Rach

Multimedia appendix 2. Search terms for PubMed, Pub Med Central, Scopus, IEEE Xplore and ACM Digital Library.

As recommended by the JBI Manual for Evidence Synthesis, the search string was constructed according to the PCC (Population/participants, Concept, and Context) framework which is shown here by example for PubMed. No specific restrictions were used to define the population. The concept was defined by terms related to automatic translation technology and the context by defining settings for population-based communication in PH, epidemiology and community-based healthcare.

For each repository, the search strings were constructed and readapted to specific syntax of their query boxes. The search was limited to abstracts, titles and full text of publications.

1. PubMed Search Strategy

PPC Criteria	Search Terms
Population<br>• No specific restrictions were used to define the population	NA
Concept<br>• MT technology and software<br>• Alternative digital information technologies used for multilingual information dissemination	(<br>"machine translation" [Title/Abstract]<br>**OR** "google translate"[Title/Abstract]<br>**OR** (<br>(bilingual*[Title/Abstract] **OR** "bi-lingual"[Title/Abstract] **OR** multilingual*[Title/Abstract] **OR** "multi-lingual"[Title/Abstract] **OR** "multiple languages"[Title/Abstract] **OR** plurilingual*[Title/Abstract])<br>**AND**<br>(app[Title/Abstract] **OR** apps[Title/Abstract] **OR** app-based[Title/Abstract] **OR** internet-based[Title/Abstract] **OR** online[Title/Abstract] **OR** platform[Title/Abstract] **OR** platforms[Title/Abstract] **OR** smartphone[Title/Abstract] **OR** smartphones[Title/Abstract] **OR** "translator"[Title/Abstract] **OR** "web application"[Title/Abstract] **OR** "web applications"[Title/Abstract] **OR** web-based[Title/Abstract] **OR** website[Title/Abstract] **OR** websites[Title/Abstract] **OR** "web service"[Title/Abstract] **OR** "web services"[Title/Abstract])<br>)<br>)
	AND
Context<br>• *Terms referring to population-based studies*<br>• Terms referring to public or collective engagement or participation in healthcare	(<br>care **OR** "Case-Control" **OR** census **OR** censuses **OR** "cohort study" **OR** "cohort studies" **OR** community **OR** consent **OR** "Cross-Sectional" **OR** engagement **OR** engaged **OR** enrollment **OR** enrolment **OR** epidemiology **OR** epidemiological **OR** health **OR** healthcare **OR** hospital **OR** questionnaire **OR** questionnaires **OR** intervention **OR** interventions **OR** material **OR** materials **OR** participant **OR** participation **OR** "population health" **OR** "public health" **OR** recruitment **OR** recruited **OR** "retention" **OR** "selection bias" **OR** "sampling bias" **OR** survey **OR** surveys **OR** trial **OR** trials **OR** volunteer **OR** voluntary **OR** "Case-Control Studies"[MeSH Terms] **OR** "Censuses"[MeSH Terms] **OR** "Cohort Studies"[MeSH Terms] **OR** "Cross-Sectional Studies"[MeSH Terms] **OR** "Epidemiologic Research Design"[MeSH Terms] **OR** "Epidemiologic Study Characteristics"[MeSH Terms] **OR** "selection bias"[MeSH Terms] **OR** "Surveys and Questionnaires"[MeSH Terms]<br>)

Note: Original search strategy
Webpage: https://pubmed.ncbi.nlm.nih.gov/

2. PubMed Central search string for Query Box

(care OR "Case-Control" OR census OR censuses OR "cohort study" OR "cohort studies" OR community OR consent OR "Cross-Sectional" OR engagement OR engaged OR enrollment OR enrolment OR epidemiology OR epidemiological OR health OR healthcare OR hospital OR questionnaire OR questionnaires OR intervention OR interventions OR material OR materials OR participant OR participation OR "population health" OR "public health" OR recruitment OR recruited OR "retention" OR "selection bias" OR "sampling bias" OR survey OR surveys OR trial OR trials OR volunteer OR voluntary OR "Case-Control Studies"[MeSH Terms] OR "Censuses"[MeSH Terms] OR "Cohort Studies"[MeSH Terms] OR "Cross-Sectional Studies"[MeSH Terms] OR "Epidemiologic Research Design"[MeSH Terms] OR "Epidemiologic Study Characteristics"[MeSH Terms] OR "selection bias"[MeSH Terms] OR "Surveys and Questionnaires"[MeSH Terms]) AND ("machine translation"[Abstract] OR "google translate"[Abstract] OR "machine translation"[Title] OR "google translate"[Title] OR ((bilingual*[Abstract] OR "bi-lingual"[Abstract] OR multilingual*[Abstract] OR "multi-lingual"[Abstract] OR "multiple languages"[Abstract] OR plurilingual*[Abstract] OR bilingual*[Title] OR "bi-lingual"[Title] OR multilingual*[Title] OR "multi-lingual"[Title] OR "multiple languages"[Title] OR plurilingual*[Title]) AND (app[Abstract] OR apps[Abstract] OR app-based[Abstract] OR internet-based[Abstract] OR online[Abstract] OR platform[Abstract] OR platforms[Abstract] OR smartphone[Abstract] OR smartphones[Abstract] OR "translator"[Abstract] OR "web application"[Abstract] OR "web applications"[Abstract] OR web-based[Abstract] OR website[Abstract] OR websites[Abstract] OR "web service"[Abstract] OR "web services"[Abstract] OR app[Title] OR apps[Title] OR app-based[Title] OR internet-based[Title] OR online[Title] OR platform[Title] OR platforms[Title] OR smartphone[Title] OR smartphones[Title] OR "translator"[Title] OR "web application"[Title] OR "web applications"[Title] OR web-based[Title] OR website[Title] OR websites[Title] OR "web service"[Title] OR "web services"[Title])))

Notes: MT Technology tuples search appear in all Titles or Abstracts at least once + PMC Syntax requires to search titles and abstracts separately
Webpage: https://www.ncbi.nlm.nih.gov/pmc?db=PMC

3. Institute of Electrical and Electronics Engineers (IEEE) Xplore search string for Query Box

("Abstract":care OR "Abstract":"Case-Control" OR "Abstract":census OR "Abstract":censuses OR "Abstract":"cohort study" OR "Abstract":"cohort studies" OR "Abstract":consent OR "Abstract":community OR "Abstract":"Cross-Sectional" OR "Abstract":engagement OR "Abstract":engaged OR "Abstract":enrollment OR "Abstract":epidemiology OR "Abstract":epidemiological OR "Abstract":health OR "Abstract":healthcare OR "Abstract":hospital OR "Abstract":intervention OR "Abstract":interventions OR "Abstract":questionnaire OR "Abstract":questionnaires OR "Abstract":material OR "Abstract":materials OR "Abstract":participant OR "Abstract":participants OR "Abstract":participation OR "Abstract":"population health" OR "Abstract":"public health" OR "Abstract":recruited OR "Abstract":recruitment OR "Abstract":recruiting OR "Abstract":"retention" OR "Abstract":"sampling bias" OR "Abstract":"selection bias" OR "Abstract":survey OR "Abstract":surveys OR "Abstract":trial OR "Abstract":trials OR "Abstract":voluntary OR "Abstract":volunteer OR "Abstract":volunteers OR "Document Title":care OR "Document Title":"Case-Control" OR "Document Title":census OR "Document Title":censuses OR "Document Title":"cohort study" OR "Document Title":"cohort studies" OR "Document Title":consent OR "Document Title":community OR "Document Title":"Cross-Sectional" OR "Document Title":engagement OR "Document Title":engaged OR "Document Title":enrollment OR "Document Title":epidemiology OR "Document Title":epidemiological OR "Document Title":health OR "Document Title":healthcare OR "Document Title":hospital OR "Document Title":intervention OR "Document Title":interventions OR "Document Title":questionnaire OR "Document Title":questionnaires OR "Document Title":material OR "Document Title":materials OR "Document Title":participant OR "Document Title":participants OR "Document Title":participation OR "Document Title":"population health" OR "Document Title":"public health" OR "Document Title":recruited OR "Document Title":recruitment OR "Document Title":recruiting OR "Document Title":"retention" OR "Document Title":"sampling bias" OR "Document Title":"selection bias" OR "Document Title":survey OR "Document Title":surveys OR "Document Title":trial OR "Document Title":trials OR "Document Title":voluntary OR "Document Title":volunteer OR "Document Title":volunteers) AND ("Abstract":"machine translation" OR "Document Title":"machine translation" OR "Abstract":"google translate" OR "Document Title":"google translate" OR (("Abstract":multilingual OR "Document Title":multilingual OR "Abstract":"multi-lingual" OR "Document Title":"multi-lingual" OR "Abstract":bilingual OR "Document Title":bilingual OR "Abstract":bi-lingual OR "Document Title":bi-lingual OR "Abstract":plurilingual OR "Document Title":plurilingual OR "Abstract":"multiple languages" OR "Document Title":"multiple languages") AND ("Abstract":app OR "Document Title":app OR "Abstract":apps OR "Document Title":apps OR "Abstract":app-based OR "Document Title":app-based OR "Abstract":internet-based OR "Document Title":internet-based OR "Abstract":online OR "Document Title":online OR "Abstract":platform OR "Document Title":platform OR "Abstract":platforms OR "Document Title":platforms OR "Abstract":smartphone OR "Document Title":smartphone OR "Abstract":smartphones OR "Document Title":smartphones OR "Abstract":translator OR "Document Title":translator OR "Abstract":"web application" OR "Document Title":"web application" OR "Abstract":web-based OR "Document Title":web-based OR "Abstract":website OR "Document Title":website OR "Abstract":websites OR "Document Title":websites OR "Abstract":"web service" OR "Document Title":"web service" OR "Abstract":"web services" OR "Document Title":"web services")))

Notes: No MESH terms available + MT Technology and healthcare tuples search appear in all Titles or Abstracts at least once
Website: https://ieeexplore.ieee.org/Xplore/home.jsp

4. Association for Computing Machinery (ACM) Digital Library search string for Query Box

(Abstract:((care* OR "Case-Control" OR census* OR "cohort study" OR "cohort studies" OR consent* OR community OR "Cross-Sectional" OR engag* OR enrollment OR epidemiolog* OR health* OR healthcare OR hospital OR intervention* OR material* OR particip* OR "population health" OR "public health" OR questionnaire* OR recruit* OR retention OR "sampling bias" OR "selection bias" OR survey* OR trial* OR volunteer*)) OR Title:((care* OR "Case-Control" OR census* OR "cohort study" OR "cohort studies" OR consent* OR community OR "Cross-Sectional" OR engag* OR enrollment OR epidemiolog* OR health* OR healthcare OR hospital OR intervention* OR material* OR particip* OR "population health" OR "public health" OR questionnaire* OR recruit* OR retention OR "sampling bias" OR "selection bias" OR survey* OR trial* OR volunteer*))) AND (Abstract:(("machine translation" OR "google translate" OR ((multilingual* OR "multi-lingual" OR bilingual* OR "bi-lingual" OR plurilingual* OR "multiple languages") AND (app OR apps OR app-based OR internet-based OR online OR platform* OR smartphone* OR "translator" OR "web-based" OR website* OR "web service")))) OR Title:(("machine translation" OR "google translate" OR ((multilingual* OR "multi-lingual" OR bilingual* OR "bi-lingual" OR plurilingual* OR polyglot OR "multiple languages") AND (app OR apps OR app-based OR internet-based OR online OR platform* OR smartphone* OR "translator" OR "web-based" OR website* OR "web service")))))

Notes: MT Technology and healthcare tuples search should appear in all Titles or Abstracts at least once
Website: https://dl.acm.org/

5. SCOPUS search string for Query Box

TITLE-ABS ((care OR "Case-Control" OR census OR censuses OR "cohort study" OR "cohort studies" OR community OR consent OR "Cross-Sectional" OR engagement OR engaged OR enrollment OR enrolment OR epidemiology OR epidemiological OR health OR healthcare OR hospital OR questionnaire OR questionnaires OR intervention OR interventions OR material OR materials OR participant OR participation OR "population health" OR "public health" OR recruitment OR recruited OR "retention" OR "selection bias" OR "sampling bias" OR survey OR surveys OR trial OR trials OR volunteer OR voluntary) AND ("machine translation" OR "google translate" OR ((multilingual* OR "multi-lingual" OR bilingual* OR "bi-lingual" OR plurilingual* OR "multiple languages") AND (app OR apps OR app-based OR internet-based OR online OR platform OR platforms OR smartphone OR smartphones OR "translator" OR "web application" OR "web applications" OR web-based OR website OR websites OR "web service" OR "web services"))))

Notes: No mesh terms + MT Technology and Healthcare Settings should appear in Titles or Abstracts at least once + Access via Bremen matriculation
Website: https://www.scopus.com/
