# Supplementary material for: The Use of Machine Translation for Outreach and Health Communication in Epidemiology and Public Health: Scoping Review
Source: JMIR Public Health Surveill. 2023 Nov 20;9:e50814. doi: 10.2196/50814 (PMC10696499; doi:10.2196/50814)
Supplement: Multimedia Appendix 3 [file publichealth_v9i1e50814_app3.pdf]

The use of machine translation for outreach and health communication in epidemiology and public health: scoping review

Paula S. Herrera-Espejel and Stefan Rach

Multimedia appendix 3. List of excluded studies during full text screening.

Retrieval Date	Authors (Year)	Study Title	doi	Exclusion Reason
31.01.2022	Bowker L. (2008)	Official language minority communities, machine translation, and translator education: Reflections on the status quo and considerations for the future	10.7202/037491ar	Ineligible study setting (does not specifically address research question)
31.01.2022	Desjardins R. (2021)	Nutrition and translation	10.4324/9781003167983-28	Ineligible study design
31.01.2022	George R. et al (2021)	Harnessing technology to respond to the global demand for learning during the COVID-19 pandemic	NA	Ineligible study setting (does not specifically address research question)
31.01.2022	Haddow B. et al (2021)	Machine translation in healthcare	10.4324/9781003167983-10	Ineligible study design
31.01.2022	Huck M. et al (2017)	LMU Munich’s neural machine translation systems for news articles and health information texts	NA	short paper of included original paper (duplicate information)
31.01.2022	Mandel H. et al (2013)	Exploring local public health workflow in the context of automated translation technologies.	NA	short paper of included original paper (duplicate information)
31.01.2022	Miller R. et al (2021)	Evaluating local multilingual health care information environments on the internet: A pilot study	10.3390/ijerph18136836	short paper of included original paper (duplicate information)
31.01.2022	Sandberg C. et al (2020)	Development of a free online interactive naming therapy for bilingual aphasia	10.1044/2019_AJSLP-19-0035	Ineligible study setting
31.01.2022	Turner A.M. et al (2015)	Machine Assisted Translation of Health Materials to Chinese: An Initial Evaluation	10.3233/978-1-61499-564-7-979	short paper of included original paper (duplicate information)
03.03.2023	Zhu Y. et al (2022)	Proposing Causal Sequence of Death by Neural Machine Translation in Public Health Informatics	10.1109/JBHI.2022.3163013	Ineligible study setting
