# Supplementary material for: The Use of Machine Translation for Outreach and Health Communication in Epidemiology and Public Health: Scoping Review
Source: JMIR Public Health Surveill. 2023 Nov 20;9:e50814. doi: 10.2196/50814 (PMC10696499; doi:10.2196/50814)

The use of machine translation for outreach and health communication in epidemiology and public health: scoping review

Paula S. Herrera-Espejel and Stefan Rach

Multimedia appendix 4. Study characteristics

- Table of 46 selected studies in the scoping review

Authors	Year	Article Title	Article Type	Journal Title	Journal Type	Publisher	DOI
Almahasees Z., Jaccomard H.	2020	Facebook translation service (FTS) usage among Jordanians during COVID-19 lockdown	Original Paper	Advances in Science, Technology and Engineering Systems	Technology & Informatics	ASTES Publishers	10.25046/aj050661
Almahasees Z., Meqdadi S., Albudairi Y.	2021	Evaluation of google translate in rendering English covid-19 texts into Arabic	Original Paper	Journal of Language and Linguistic Studies	Life Sciences & Biomedicine	Selcuk University	10.52462/jlls.149
Anazawa R., Ishikawa H., Park M., Kiuchi T.	2012	Preliminary study of online machine translation use of nursing literature: Quality evaluation and perceived usability	Original Paper	BMC Research Notes	Life Sciences & Biomedicine	NA[1]	10.1186/1756-0500-5-635
Anazawa R, Ishikawa H, Park MJ, Kiuchi T.	2013	Online machine translation use with nursing literature: evaluation method and usability	Original Paper	Computers Informatics Nursing	Health Technology & Informatics	NA	10.1097/NXN.0b013e3182701056
Anazawa R., Ishikawa H., Takahiro K.	2013	Use of online machine translation for nursing literature: A questionnaire-based survey	Original Paper	Open Nursing Journal	Life Sciences & Biomedicine	NA	10.2174/1874434601307010022
Anazawa R., Ishikawa H., Takahiro K.	2013	Evaluation of online machine translation by nursing users	Original Paper	Computers Informatics Nursing	Health Technology & Informatics	NA	10.1097/NXN.0b013e3182999dc2
Bedrick S.D., Mauro A.	2009	A Multi-Lingual Web Service for Drug Side-Effect Data	Conference Paper	American Medical Informatics Association Annual Symposium Proceedings	Health Technology & Informatics	NA	NA
Capurro D., Chaudhuri S., Turner A.M.	2015	The Online Availability of Multilingual Health Promotion Materials Produced by Local Health Departments: An Information Assessment	Original Paper	Studies in Health Technology and Informatics	Health Technology & Informatics	IOS Press	10.3233/978-1-61499-564-7-380
Chen X., Acosta S., Barry A.E.	2016	Evaluating the accuracy of google translate for diabetes education material	Original Paper	Journal of Medical Internet Research Diabetes	Health Technology & Informatics	JMIR Publications Inc.	10.2196/diabetes.5848
Cornelison B.R., Al-Mohaish S., Sun Y., Edwards C.J.	2021	Accuracy of Google Translate in translating the directions and counseling points for top-selling drugs from English to Arabic, Chinese, and Spanish	Original Paper	American Journal of Health-System Pharmacy	Life Sciences & Biomedicine	NA	10.1093/ajhp/zxab224

Das P., Kuznetsova A., Zhu M., Milanaik R.	2019	Dangers of Machine Translation: The Need for Professionally Translated Anticipatory Guidance Resources for Limited English Proficiency Caregivers	Original Paper	Clinical Pediatrics (Phila)	Life Sciences & Biomedicine	NA	10.1177/0009922818809494
Dew K., Turner A.M., Desai L., Martin N., Laurenzi A., Kirchhoff K.	2015	PHAST: A Collaborative Machine Translation and Post-Editing Tool for Public Health	Conference Paper	American Medical Informatics Association Annual Symposium Proceedings	Health Technology & Informatics	NA	NA
Dharmawan R., Nababan M.R., Tarjana M.S.S., Djatmika D.	2019	Mistranslation and maltranslation in a medical website: Evidences from Dorland's medical dictionary	Original Paper	Journal of Social Studies Education Research	Life Sciences & Biomedicine	Association for Social Studies Educa	NA
Dumitran A.	2021	Translation Error in Google Translate from English Into Romanian In Texts Related to Coronavirus	Conference Paper	eLearning and Software for Education Conference	Technology & Informatics	National Defence University - Carol I Printing House	NA
Guo JW.	2016	Is Google Translate Adequate for Facilitating Instrument Translation from English to Mandarin?	Original Paper	Computers Informatics Nursing	Health Technology & Informatics	NA	10.1097/CIN.0000000000000287
Khanna R.R., Karliner L.S., Eck M., Vittinghoff E., Koenig C.J., Fang M.C.	2011	Performance of an online translation tool when applied to patient educational material	Original Paper	Journal of Hospital Medicine	Life Sciences & Biomedicine	NA	10.1002/jhm.898
Khoong E.C., Steinbrook E., Brown C., Fernandez A.	2019	Assessing the Use of Google Translate for Spanish and Chinese Translations of Emergency Department Discharge Instructions	Original Paper	JAMA Intern Med	Life Sciences & Biomedicine	NA	10.1001/jamainternmed.2018.7653
Kirchhoff K., Turner A.M., Axelrod A., Saavedra F.	2011	Application of statistical machine translation to public health information: A feasibility study	Original Paper	American Medical Informatics Association Annual Symposium Proceedings	Health Technology & Informatics	NA	10.1136/amiajnl-2011-000176
Laurenzi A., Brownstein M., Turner A.M., Kientz J.A., Kirchhoff K.	2013	A Web-Based Collaborative Translation Management System for Public Health Workers	Conference Paper	Conference on Human Factors in Computing Systems - Proceedings	Technology & Informatics	Association for Computing Machinery	10.1145/2468356.2468446
Li J., Lester C., Zhao X., Ding Y., Jiang Y., Vinod Vydiswaran V.G.	2020	PharmMT: A neural machine translation approach to simplify prescription directions	Conference Paper	Findings of the Association for Computational Linguistics Findings of ACL: EMNLP 2020	Technology & Informatics	Association for Computational Linguistics (ACL)	NA
Liang Y., Han W.	2022	Source text pre-editing versus target text post-editing in using Google Translate to provide health services to culturally and linguistically diverse clients	Original Paper	Science, Engineering and Health Studies	Health Technology & Informatics	Silpakorn University	10.14456/sehs.2022.25
Liu W., Cai S., Ramesh B.P., Chiriboga G., Knight K., Yu H.	2015	Translating Electronic Health Record Notes from English to Spanish: A Preliminary Study	Conference Paper	ACL-IJCNLP 2015 - BioNLP 2015: Workshop on Biomedical Natural Language	Technology & Informatics	Association for Computational Linguistics (ACL)	NA

				Processing, Proceedings of the Workshop			
Mahadin D.K., Olimat S.N.	2022	Jordanian Translators' Use of Machine Translation and Glossary of COVID-19 Terminology with Reference to Arabic	Original Paper	New Voices in Translation Studies	Health Technology & Informatics	International Association of Translation and Intercultural Studies	NA
Mandel H., Turner A.M.	2013	Exploring local public health work in the context of novel translation technologies	Conference Paper	Studies in Health Technology and Informatics	Health Technology & Informatics	IOS Press	10.3233/978-1-61499-289-9-1209
Miller J.M., Harvey E.M., Bedrick S., Mohan P., Calhoun E.	2018	Simple patient care instructions translate best: Safety guidelines for physician use of Google translate	Original Paper	Journal of Clinical Outcomes Management	Life Sciences & Biomedicine	Turner White Communications Inc.	NA
Pandey R., Gautam V., Pal R., Sethi, T.	2022	A machine learning application for raising WASH awareness in the times of COVID-19 pandemic	Original Paper	Scientific Reports	Life Sciences & Biomedicine	Nature Research	10.1038/s41598-021-03869-6
Patil S., Davies P.	2014	Use of Google Translate in medical communication: evaluation of accuracy	Original Paper	British medical journal	Life Sciences & Biomedicine	NA	10.1136/bmj.g7392
Pecina P., Dušek O., Goeuriot L., Hajič J., Hlaváčová J., Jones G.J., Kelly L., Leveling J., Mareček D., Novák M., Popel M., Rosa R., Tamchyna A., Urešová Z.	2014	Adaptation of machine translation for multilingual information retrieval in the medical domain	Original Paper	Artificial Intelligence in Medicine	Health Technology & Informatics	NA	10.1016/j.artmed.2014.01.004
Rodriguez J.A., Davis R.B., Percac-Lima S.	2019	Non-English Language Availability of Community Health Center Websites	Original Paper	Medical Care	Life Sciences & Biomedicine	Lippincott Williams and Wilkins	10.1097/MLR.000000000000001027
Skianis K., Briand Y., Desgrippes F.	2020	Evaluation of machine translation methods applied to medical terminologies	Conference Paper	EMNLP 2020 - 11th International Workshop on Health Text Mining and Information Analysis, LOUHI 2020, Proceedings of the Workshop	Technology & Informatics	Association for Computational Linguistics (ACL)	10.18653/v1/2020.louhi-1.7
Taira B.R., Kreger V., Orue A., Diamond L.C.	2021	A Pragmatic Assessment of Google Translate for Emergency Department Instructions	Original Paper	Journal of General Internal Medicine	Life Sciences & Biomedicine	Springer	10.1007/s11606-021-06666-z
Takakusagi Y., Oike T., Shirai K., Sato H., Kano K., Shima S., Tsuchida K., Mizoguchi N., Serizawa I., Yoshida D., Kamada T., Katoh H.	2021	Validation of the Reliability of Machine Translation for a Medical Article from Japanese to English Using DeepL Translator	Original Paper	Cureus Journal of Medical Science	Life Sciences & Biomedicine	NA	10.7759/cureus.17778

Taylor R.M., Crichton N., Moult B., Gibson F.	2015	A prospective observational study of machine translation software to overcome the challenge of including ethnic diversity in healthcare research	Original Paper	Nursing Open	Life Sciences & Biomedicine	Blackwell Publishing Ltd	10.1002/nop2.13
Tensmeyer N.C., Dinh N.N.L., Sun L.T., Meyer C.B.	2022	Analysis of Language Translations of State Governments' Coronavirus Disease 2019 Vaccine Websites	Original Paper	Health Equity	Life Sciences & Biomedicine	Mary Ann Liebert Inc.	10.1089/heq.2021.0189
Turner A.M., Mandel H., Capurro D.	2013	Local health department translation processes: potential of machine translation technologies to help meet needs.	Conference Paper	American Medical Informatics Association Annual Symposium Proceedings	Health Technology & Informatics	NA	NA
Turner A.M., Bergman M., Brownstein M., Cole K., Kirchhoff K.	2014	A comparison of human and machine translation of health promotion materials for public health practice: Time, costs, and quality	Original Paper	Journal of Public Health Management and Practice	Life Sciences & Biomedicine	Lippincott Williams and Wilkins	10.1097/PHH.0b013e3182a95c87
Turner A.M., Brownstein M.K., Cole K., Karasz H., Kirchhoff K.	2015	Modeling workflow to design machine translation applications for public health practice	Original Paper	Studies in Health Technology and Informatics	Health Technology & Informatics	Academic Press Inc.	10.1016/j.jbi.2014.10.005
Turner A.M., Dew K.N., Desai L., Martin N., Kirchhoff K.	2015	Machine translation of public health materials from English to Chinese: A feasibility study	Original Paper	JMIR Public Health and Surveillance	Life Sciences & Biomedicine	JMIR Publications Inc.	10.2196/publichealth.4779
Way A., Haque R., Xie G., Gaspari F., Popović M., Poncelas A.	2020	Rapid development of competitive translation engines for access to multilingual covid-19 information	Original Paper	Journal of Informatics	Technology & Informatics	MDPI Multidisciplinary Digital Publishing Institute	10.3390/informatics7020019
Wu C., Xia F., Deleger L., Solti I.	2011	Statistical Machine Translation for Biomedical Text: Are We There Yet?	Conference Paper	American Medical Informatics Association Annual Symposium Proceedings	Health Technology & Informatics	NA	NA
Xie W., Ji M., Huang R., Hao T., Chow C.Y.	2021	Predicting risks of machine translations of public health resources by developing interpretable machine learning classifiers	Original Paper	International Journal of Environmental Research and Public Health	Life Sciences & Biomedicine	MDPI AG	10.3390/ijerph18168789
Yan R., Liao W., Cui J., Zhang H., Hu Y., Zhao D.	2021	Multilingual COVID-QA: Learning towards global information sharing via web question answering in multiple languages	Conference Paper	The Web Conference 2021 - Proceedings of the World Wide Web Conference, WWW 2021	Technology & Informatics	Association for Computing Machinery, Inc	10.1145/3442381.3449991
Yepes A.J., Névéol A., Neves M., Verspoor K., Bojar O., Boyer A., Grozea C., Haddow B., Kittner M., Lichtblau Y., Pecina P., Roller	2017	Findings of the WMT 2017 biomedical translation shared task	Conference Paper	WMT 2017 - 2nd Conference on Machine Translation, Proceedings	Technology & Informatics	Association for Computational Linguistics (ACL)	NA

R., Rosa R., Siu A., Thomas P., Trescher S.							
Yang L.W.Y., Ng W.Y., Lei X., Tan S.C.Y., Wang Z., Yan M., Pargi M.K., Zhang X., Lim J.S., Gunasekeran D.V., Tan F.C.P., Lee C.E., Yeo K.K., Tan H.K., Ho H.S.S., Tan B.W.B., Wong T.Y., Kwek K.Y.C., Goh R.S.M., Liu Y., Ting D.S.W.	2023	Development and testing of a multi-lingual Natural Language Processing-based deep learning system in 10 languages for COVID-19 pandemic crisis: A multi-center study	Original Paper	Front Public Health	Life Sciences & Biomedicine	Front Public Health.	10.3389/fpubh.2023.1063466
Zeng-Treitler Q., Kim H., Rosemblat G., Keselman A.	2010	Can multilingual machine translation help make medical record content more comprehensible to patients?	Original Paper	Studies in Health Technology and Informatics	Health Technology & Informatics	IOS Press	10.3233/978-1-60750-588-4-73
Ziganshina L.E., Yudina E.V., Gabdrakhmanov A.I., Ried J.	2021	Assessing human post-editing efforts to compare the performance of three machine translation engines for English to Russian translation of Cochrane plain language health information: Results of a randomized comparison	Original Paper	Journal of Informatics	Technology & Informatics	MDPI AG	10.3390/informatics8010009

[1] The following abbreviation "NA" stands for "Not available".

- <u>Distribution of selected papers by publication type and year (from 2007 to 2023)</u>

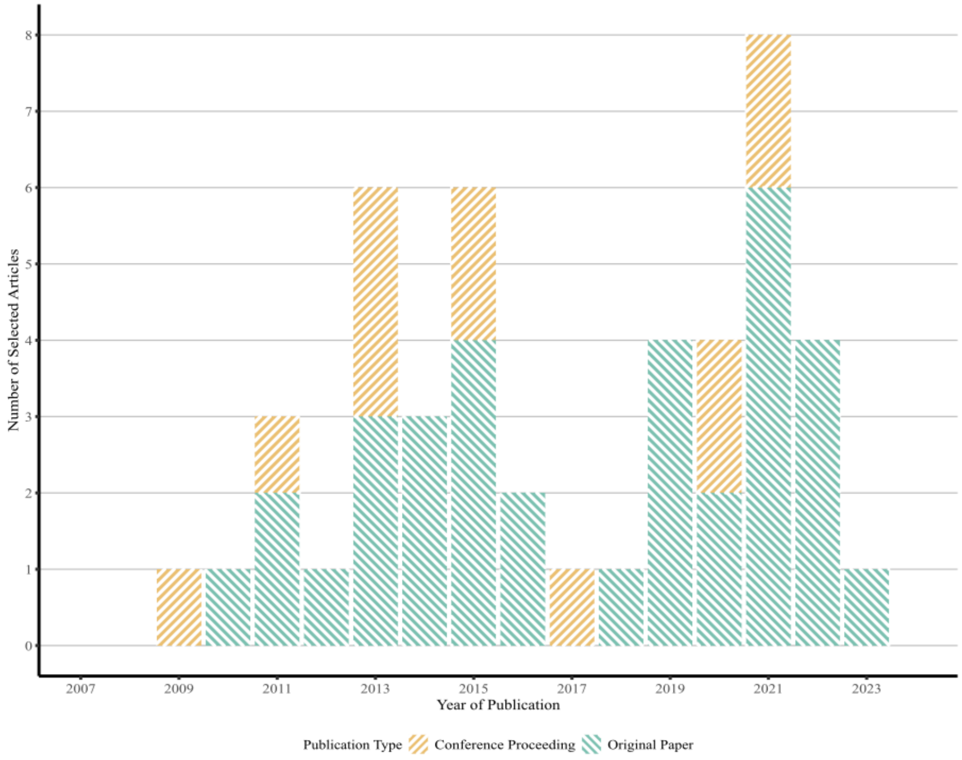

Supplement: Multimedia Appendix 4 [file publichealth_v9i1e50814_app4.pdf]
