# Supplementary material for: The Use of Machine Translation for Outreach and Health Communication in Epidemiology and Public Health: Scoping Review
Source: JMIR Public Health Surveill. 2023 Nov 20;9:e50814. doi: 10.2196/50814 (PMC10696499; doi:10.2196/50814)
Supplement: Multimedia Appendix 5 [file publichealth_v9i1e50814_app5.pdf]

The use of machine translation for outreach and health communication in epidemiology and public health: scoping review

Paula S. Herrera-Espejel and Stefan Rach

Multimedia appendix 5. Transmitters, receivers, public health material, machine translation engines.

	Public Health Communication Agents			Objects of Translation				Machine Translation Technology		
**Author Item**	**Geolocation**	**Transmitter**	**Receiver**	**Material**	**Domain Type**	**Tested Source Language(s)**	**Tested Target Language(s)**	**Supervision**	**Engine Type**	**Software**
Almahasees and Jaccomard	Jordan	International & National Health Services	General public on the world wide web	Guidelines & Educational resources	domain-agnostic	English	Arabic	Unsupervised	NMT	off-the-shelf
Almahasees et al	Jordan	International & National Health Services	General public on the world wide web	Guidelines & Educational resources	domain-agnostic	English	Arabic	Unsupervised	NMT	off-the-shelf
Anazawa et al	Japan	Clinical Staff	Clinical staff (r)	Academic Research	domain-agnostic	English; Japanese	Japanese, Korean	Unsupervised	SMT	off-the-shelf
Anazawa et al	Japan	Clinical Staff	Clinical staff (r)	Academic Research	domain-agnostic	English	Japanese	Unsupervised	SMT	off-the-shelf
Anazawa et al	Japan	Clinical Staff	Clinical staff (r)	Academic Research	domain-agnostic	NS	NS	Unsupervised	SMT	off-the-shelf
Anazawa et al	Japan	Clinical Staff	Clinical staff (r)	Academic Research	domain-agnostic	NS	NS	Unsupervised	SMT	off-the-shelf
Bedrick et al	Not specified	Developers	General public offline	Simplified Medical Information	domain-specific	English	Spanish	Unsupervised	SMT	off-the-shelf
Capurro et al	USA; Washington State	Public Health Staff	General public on the world wide web	Promotion materials	NS[1]	NA	NA	NA	SMT	off-the-shelf
Chen et al	USA	Public Health Staff	Target patient populations	Promotion materials	domain-agnostic	English	Spanish, Chinese	Unsupervised	NMT	off-the-shelf
Cornelison et al	USA	Clinical Staff	General public offline	Instructions Handouts	domain-agnostic	English	Spanish, Chinese, Arabic	Unsupervised	SMT	off-the-shelf
Das et al	USA	Public Health Staff	Target patient populations	Instructions Handouts	domain-agnostic	English	Spanish, Arabic, Bengali, Chinese, French, German, Greek, Haitian, Creole, Hebrew, Hindi, Italian, Japanese, Korean, Polish, Portuguese,	Unsupervised	NMT	off-the-shelf

							Punjabi, Russian, Tagalog, Urdu, Vietnamese			
Dew et al	USA	Public Health Staff	General public offline	Guidelines & Educational resources	domain-specific	English	NS	Supervised – TTPE[3]	SMT	off-the-shelf
Dharmawan et al	Indonesia	Public Health Staff	Clinical staff (r)	Simplified Medical Information	domain-agnostic	English	Indonesian	Unsupervised	NMT	off-the-shelf
Dumitran	Not specified	International & National Health Services	General public on the world wide web	Simplified Medical Information	domain-agnostic	English	Romanian	Unsupervised	NMT	off-the-shelf
Guo	USA	Public Health Staff	Public Health Researchers (r)	Survey Instrument	domain-agnostic	English	Chinese (Manderin)	Unsupervised	NMT	off-the-shelf
Khanna et al	USA	International & National Health Services	Target patient populations	Instructions Handouts	domain-agnostic	English	Spanish	Unsupervised	SMT	off-the-shelf
Khoong et al	Not specified	Clinical Staff	General public offline	Instructions Handouts	domain-agnostic	English	Spanish, Chinese	Unsupervised	NMT	off-the-shelf
Kirchhoff et al	USA	Public Health Staff	General public offline	Promotion materials	domain-agnostic	English	Mexican-Spanish	Supervised - TTPE	SMT	off-the-shelf
Laurenzi 2013	USA	Public Health Staff	Public Health Researchers (r)	Promotion materials	domain-agnostic	English	Spanish, Russian, Vietnamese	Supervised - TTPE	SMT	off-the-shelf
Li et al	USA	Clinical Staff	Target patient populations	Simplified Medical Information	domain-specific	English physician-authored directions	English patient-facing text authored by pharmacists	Supervised – STPE[4]	NMT	in-house
Liang and Han	Australia	Public Health Staff	General public offline	Guidelines & Educational resources	domain-agnostic	English	Chinese (Manderin)	Supervised - STPE	NMT	off-the-shelf
Liu et al	USA	Public Health Staff	Target patient populations	Simplified Medical Information	domain-agnostic	English physician-authored directions	English patient-facing text	Unsupervised	SMT	in-house
Mahadin and Olimat	Jordan	International & National Health Services	General public offline	Promotion materials	domain-agnostic	NA	NA	NA	SMT	off-the-shelf
Mandel and Turner	USA; Washington State	Public Health Staff	General public offline	Promotion materials	NA[2]	NA	NA	NA	NS	NA
Miller et al	USA	Clinical Staff	Target patient populations	Instructions Handouts	domain-agnostic	English	Spanish	Supervised – IBT[5]	NMT	off-the-shelf
Pandey et al	Global	Developers	General public offline	Guidelines & Educational resources	NS	English	Hindi, vernaculars	Unsupervised	NMT	off-the-shelf
Patil and Davies	UK; Nottingham	Clinical Staff	General public offline	Simplified Medical Information	domain-agnostic	English	NS	Unsupervised	SMT	off-the-shelf

Pecina et al	USA	Clinical Staff	Target patient populations	Simplified Medical Information	domain-specific	Czech, German, French	English	Unsupervised	SMT	in-house
Rodríguez et al	USA	Public Health Staff	General public on the world wide web	Guidelines & Educational resources	NA	NA	NA	NA	NS	NA
Skianis et al	Global	Clinical Staff	Clinical staff (r)	Simplified Medical Information	domain-specific	English	French	Unsupervised	NMT	in-house
Taira et al	USA	Clinical Staff	Target patient populations	Instructions Handouts	domain-agnostic	English	Spanish, Chinese, Vietnamese, Tagalog, Korean, Armenian, Farsi	Unsupervised	NMT	off-the-shelf
Takakusagi et al	Japan	Public Health Staff	Public Health Researchers (r)	Academic Research	domain-agnostic	Japanese	English	Unsupervised	NMT	off-the-shelf
Taylor et al	UK; England, Wales	Public Health Staff	General public offline	Survey Instrument	domain-agnostic	English	Arabic, Bulgarian, Chinese(simple), Czech, Danish, Dutch, French, German, Hebrew, Hungarian, Italian, Japanese, Korean, Polish, Portuguese, Romanian, Russian, Serbian, Spanish, Swedish, Thai, Turkish, Ukrainian, Norwegian	Supervised - IBT	NMT	off-the-shelf
Tensmeyer et al	USA	Public Health Staff	General public on the world wide web	Guidelines & Educational resources	NA	NA	NA	NA	NS	NA
Turner et al	USA; Washington, Oregon, Idaho and Alaska	Public Health Staff	General public offline	Promotion materials	NA	NA	NA	NA	NS	NA
Turner et al	USA	Public Health Staff	General public offline	Promotion materials	domain-agnostic	English	Spanish	Supervised - TTPE	SMT	off-the-shelf
Turner et al	USA	Public Health Staff	General public offline	Promotion materials	domain-agnostic	NA	NA	NA	SMT	in-house
Turner et al	USA	Public Health Staff	General public offline	Promotion materials	domain-agnostic	English	Chinese	Supervised - TTPE	SMT	off-the-shelf
Way et al	Germany, Spain, UK,	Clinical Staff	General public on the	Guidelines &	domain-specific	French, Italian, German,	English; French, Italian,	Unsupervised	NMT	in-house

	France, US, Italy		world wide web	Educational resources		Spanish; English	German, Spanish			
Wu et al	USA	Public Health Staff	General public offline	Guidelines & Educational resources	domain-agnostic	English; French, Hungarian, Polish, Spanish, German, Turkish	French, Hungarian, Polish, Spanish, German, Turkish; English	Unsupervised	SMT	in-house
Xie et al	Not specified	Clinical Staff	Target patient populations	Simplified Medical Information	domain-agnostic	English	Chinese (Manderin)	Unsupervised	NMT	off-the-shelf
Yan et al	Global	International & National Health Services	General public on the world wide web	Guidelines & Educational resources	domain-specific	English	Chinese, Japanese	Unsupervised	NMT	in-house
Yang et al	Global	International & National Health Services	Clinical staff (r)	Guidelines & Educational resources	domain-agnostic	English	Chinese, Malay, Tamil, Filipino, Thai, Japanese, French, Spanish, and Portuguese.	Unsupervised	NMT	off-the-shelf
Yepes et al	Not specified	International & National Health Services	Public Health Researchers (r)	Academic Research	domain-specific	English; French, Spanish, Portuguese	Czech, German, French, Hungarian, Polish, Portuguese, Spanish, Romanian, Swedish; English	Unsupervised	NMT	in-house
Zeng-Treitler et al	USA	Clinical Staff	Target patient populations	Simplified Medical Information	domain-agnostic	English	Chinese, Korean, Spanish, Russian	Unsupervised	SMT	off-the-shelf
Ziganshina et al	Global	Public Health Staff	General public offline	Simplified Medical Information	domain-agnostic	NS	Russian	Supervised - TTPE	NMT	off-the-shelf

Note: The following abbreviations 1. “NS” stands for “Not specified”, 2. “NA” for “Not Applicable”, 3. TTPE for “Target Text Posttranslation Editing”, 4. STPE for “Source Text Pretranslation Editing”, and 5. “IBT” for “Iterative Back Translation”.
