# Supplementary material for: The Use of Machine Translation for Outreach and Health Communication in Epidemiology and Public Health: Scoping Review
Source: JMIR Public Health Surveill. 2023 Nov 20;9:e50814. doi: 10.2196/50814 (PMC10696499; doi:10.2196/50814)
Supplement: Multimedia Appendix 6 [file publichealth_v9i1e50814_app6.pdf]

The use of machine translation for outreach and health communication in epidemiology and public health: scoping review

Paula S. Herrera-Espejel and Stefan Rach

Multimedia appendix 6. Research study designs

MONITORING STUDIES (n=34)

Studies type I - MT technology assessment (n=23)

Author.Item	Identified Research Objective	Section - Page
Almahasees2021	• To assess whether Google Translate can be used to translate English COVID-19 texts into Arabic.	Methodology – p. 2070
Anazawa2012	• To "examine the quality of Google Translate (English to Japanese and Korean to Japanese)" and “the perceived usability and current use of online MT among Japanese nurses".<br>• To “examine and discuss the feasibility of online MT technology use by nursing professionals to obtain technical information from articles written in an unfamiliar foreign language.”	Abstract – p. 1<br>Introduction – p.2
Anazawa2013a	• To examine “existing methods for evaluating online machine translation quality for structural accuracy and intelligibility of translations of nursing literature”, and to discuss “the usability of machine translations”.<br>• To evaluate “the quality of MTs from English into Japanese via online MT systems".	Abstract – p.59<br>Machine Translation – p. 60
Anazawa2013c	• To survey "respondents’ impressions on the perceived usefulness of the translated abstract as a whole".<br>• To "examine and describe the evaluated GT performance for nursing literature by nursing users (quality rating by nurses)".	Abstract – p. 382<br>Research Questions – p. 383
Bedrick2009	• To describe a web service “that provides drug side-effect information from a curated public source in a machine-consumable format”<br>• To “evaluate the quality of the translations that Google’s translation tools provide to our system.”	Introduction – p.34<br>Translation Evaluation – p.36
Chen2016	• To "to evaluate the feasibility and accuracy of the Google Translate website as a tool to help LEP persons understand chronic condition management and prevention strategies” published online.	Introduction – p.2
Cornelison2021	• To evaluate “the accuracy of Google Translate in translating the most common directions and counseling points for the top 100 drugs dispensed in 2018, as identified by the ClinCalc app” (incl. English to Arabic, simplified Chinese, and Spanish).	Introduction – p.2054
Das2019	• To assess “the accuracy of a popular free machine translation service, in translating AAP anticipatory guidance safety guidelines for the top 20 foreign languages spoken in the United States."	Introduction – p.1
Dharmawan2019	• To evaluate whether Google Translate is useful to avoid mistranslation of medical terms used in different practice and research settings.	Research Design – p.224
Dumitran2021	• To conduct an “error analysis in the performance of translating (from English into Romanian) by MT, focusing on lexical and semantic errors found in texts with official information about the vaccines against coronavirus that are translated by Google Translate.”	Introduction – p.38
Guo2016	• To assess whether "the quality of translation from English to Mandarin of Google Translate is sufficient for its use in health survey instrument translation."	Section 3 – p.378
Khanna2011	• To evaluate the accuracy of Google Translate free online services for producing patient educational material in Spanish.	Introduction – p.520
Khoong2019	• To “assess the use of GT to translate emergency department (ED) discharge instructions into Spanish and Chinese."	Introduction – p.580
Kirchhoff2011	• To evaluate the performance of “a generic, state- of-the-art SMT system (i.e. Google Translate) followed by human postediting to replace the step of human only translation in the standard workflow of producing public health materials for LEP audiences”.	Introduction – p.474

Liang2022	• To assess "the efficacy of source text pre-editing (STPE) and MT to provide unbiased and effective health services to CALD clients in the health sector."<br>• To evaluate whether "STPE outperforms TTPE (target text post-editing) in using Google Translate to provide health services to culturally and linguistically diverse clients."	Introduction – p.2
Patil2014	• To "evaluate the accuracy and usefulness of Google Translate in translating common English medical statements."	Introduction – p.52
Taira2021	• To "perform a pragmatic assessment of the accuracy of GT for the written translation of commonly used ED discharge instructions given to patients in each of the most common languages spoken by LEP patients as assessed by bilingual community members".<br>• To "compare the performance of GT between languages" (i.e. Spanish, Chinese and English)	Introduction – p. 3362
Takakusagi2021	• To investigate "the accuracy of machine translation from Japanese to English for a medical article using the DeepL Translator."	Introduction – p.2
Taylor2015	• To "evaluate the quality of back translation into English of validated translations of a validated questionnaire using machine translators."<br>• To "evaluate the quality of the translation of a participant information sheet from English into other languages using machine translators."	Methods-Design – p.16
Turner2014	• To "identify the time and costs associated with human translation (HT) of public health documents".<br>• To "determine the time necessary for human postediting of MT" and "compare the quality of postedited MT and HT.	Abstract – p.1
Turner2015b	• To investigate "the types of errors in English-to-Chinese machine translations, PE time to correct them and quality of MT plus PE compared to manual translation."	Introduction – p.2
Zeng-Treitler2010	• To evaluate "whether the use of unedited MT output represents a viable option for translating electronic health records"	Background – p.74
Ziganshina2021	• To "compare machine translation engines by measuring quantitative human post-editing efforts in an established translation workflow and quality assurance process using a randomized study design."	Introduction – p.3

Studies type II – Standards or Criteria for MT quality assurance (n=3)

Author.Item	Identified Research Objective	Section - Page
Miller2018	• To determine predictors of quality and safety of machine translation (Google Translate) of patient care instructions (PCIs) and whether machine back translation is useful in quality assessment.	Introduction – p.19
Skianis2020	• To "present our evaluation work of the latest machine translation techniques addressing medical terminologies."<br>• To "investigate existing machine translation research studies concerning medical terms and documents, with a comparison of the relative methods."	Abstract – p.1<br>Introduction – p.1
Xie2021	• To "develop machine learning classifiers as a risk-prevention mechanism and "to predict the likelihood of clinically significant mistakes or incomprehensible MT outputs based on the features of English source information as input to the MT systems"<br>• To "explore the risks of MT tools in the translation of online health promotion resources for non-English speaking readers" and to classify types of MT errors which are clinically significant.	Introduction – p.2<br>Related work – p.2

Studies type III - MT tool development or design (n=8)

Author.Item	Identified Research Objective	Section - Page
Li2020	• To report on the development of a "machine translation- based approach, called PharmMT, to simplify e- prescription directions authored by physicians into patient-friendly language."	Introduction – p.2785
Liu2015	• To report on the development of "an EHR domain– specific English-Spanish MT system called NoteAidSpanish, which may help over 37 million Spanish speaking US residents to meaningfully use their EHRs."	Introduction – p. 134
Pecina2014	• To "investigate machine translation (MT) of user search queries in the context of cross-lingual information retrieval (IR) in the medical domain."<br>• To report on the development of their MT system used to produce best possible translation queries	Abstract – p.165<br>Introduction – p.166

Way2020	• To report on the development of “a range of MT systems to facilitate access to multilingual content related to COVID-19” information”.	Introduction and Motivation – p. 2
Wu2011	• To report on the development of an “in-house system” and to evaluate “its output for each translation pair on large scale both with automated BLEU scores and human judgment”.	Abstract – p.1290
Yan2021	• To “propose a multilingual COVID-QA model to answer people’s questions in their own languages while the model is able to absorb knowledge from other languages”<br>• To develop a “framework which incorporates (unsupervised) translation alignment to learn as pseudo-parallel data.”	Abstract – p. 2590
Yang2023	• To report on the development of “a multi-lingual chatbot able to respond accurately and quickly to general COVID-19 related questions by patients and the public.”	Introduction – p.2
Yepes2017	• To report on the performance of their in-house MT systems from English to several other languages.	Introduction – p.234-235

EVALUATION STUDIES (n=12)

Studies type IV - End-user needs and attitudes identification (n=4)

Author.Item	Identified Research Objective	Section - Page
Almahasees2020	• To provide "constructive feedback about whether FTS is considered a reliable source of information or not and the degree of trust among the end-users"	Introduction – p. 515
Anazawa2013b	• To "explore how Japanese nursing professionals use online MT and perceive its usability in reading English articles"<br>• To "discuss what should be considered for better utilization of online MT lessening the language barrier".	Aim – p. 23
Mahadin2022	• To “investigate Jordanian translators’ views on the use of MT for translating COVID-19 related terms with reference to Arabic” and their “use of COVID-19 glossaries to enhance the quality and accuracy of their translation”.<br>• To review “translators’ perspectives on the use of translation resources and tools within crisis situations, and provides a critical evaluation of relevant studies on MT with reference to Arabic".	Abstract – p.25<br>Introduction – p.27
Mandel2013	• To explore “the role of technology in translation and document exchange in public health practice and workflow processes”.	Introduction – p.1209

Studies type V - Economic evaluations (n=4)

Author.Item	Identified Research Objective	Section - Page
Capurro2015	• To determine “the availability of manually translated documents in public health promotion” and to “assess whether LHDs were meeting the LEP health information needs of their communities."	Introduction – p.381
Rodríguez2019	• To determine “the language availability of HRSA-supported CHC websites and assess its association with community demographics”.	Introduction – p.122
Tensmeyer2022	• To conduct “a comprehensive analysis of state COVID-19 websites for availability of professionally translated information about the COVID-19 vaccine and how to obtain it”.<br>• To evaluate “how state-provided translations compared with languages spoken by their respective populations based on American Community Survey (ACS) data on language use".	Introduction – p.739
Turner2013	• To explore “barriers to translation of health information” and “new technologies to improve access to multilingual public health materials”<br>• To explore “current translation practices, and assessed the attitudes of health department directors and translators towards technologies, including machine translation, to assist with creation and sharing of translated health promotion materials for LEP groups”.	Introduction – p.1378<br>Background – p.1379

Studies type VI - Implementation research (n=4)

Author.Item	Identified Research Objective	Section - Page
Dew2015	• To describe a "collaborative machine translation (MT) plus post-editing system called PHAST (Public Health Automatic System for Translation, phastsystem.org)".	Abstract – p.492

	• To report on the methods used for the “design and evaluation of PHAST, a collaborative translation tool developed to facilitate the integration of MT into the typical workflow of public health practice (phastsystem.org).”	Introduction and Background – p.492
Laurenzi2013	• To report on the design process (incl. usability testing and design iteration) and development of “a software tool to facilitate the integration of MT into the typical workflow of a public health employee”.<br>• To describe “the initial prototype design and implementation of such a tool, with a focus on applications in public health departments” (i.e. a web-based prototype collaborative translation management system”).	Introduction – p.512
Pandey2022	• To assess “the use of neural models for text summarization and machine learning for delivering WHO matched COVID-19 information”.<br>• To develop a “symptom assessment tool and segmentation insights for improving the delivery of information”.	Abstract – p.1
Turner2015a	• To “provide a detailed understanding of the information workflow processes related to translating health promotion materials for limited English proficiency individuals in order to inform the design of context-driven machine translation (MT) tools for public health (PH)".<br>• To explore “current PH translation processes to better understand how MT technology could successfully be applied to PH settings” (incl. “workflows studies …to identify functional requirements for engineering software designed to improve the production of multilingual health education materials for PH practice”).	Abstract – p. 136<br>Introduction – p.137
