# Supplementary material for: The Use of Machine Translation for Outreach and Health Communication in Epidemiology and Public Health: Scoping Review
Source: JMIR Public Health Surveill. 2023 Nov 20;9:e50814. doi: 10.2196/50814 (PMC10696499; doi:10.2196/50814)
Supplement: Multimedia Appendix 7 [file publichealth_v9i1e50814_app7.pdf]

The use of machine translation for outreach and health communication in epidemiology and public health: scoping review

Paula S. Herrera-Espejel and Stefan Rach

Multimedia Appendix 7. Authors' sentiment on principle findings in discussions and conclusions.

Author Item	TR[1] Score	SER[2] Score	ELR[3] Score
Almahasees2020	Neutral	Positive	NA
Almahasees2021	Negative	NA	NA
Anazawa2012	Neutral	Neutral	NA
Anazawa2013a	Neutral	Neutral	NA
Anazawa2013b	NA[4]	Positive	NA
Anazawa2013c	Neutral	Neutral	NA
Bedrick2009	Neutral	NA	NA
Liu2015	NA	Neutral	NA
Liang2022	NA	Positive	NA
Cornelison2021	Neutral	NA	Neutral
Capurro2015	Neutral	NA	Negative
Chen2016	Neutral	NA	NA
Das2019	Neutral	NA	Negative
Dumitran2021	Negative	NA	NA
Dew2015	Neutral	NA	NA
Khanna2011	Positive	Positive	NA
Khoong2019	Neutral	NA	Neutral
Kirchhoff2011	Positive	NA	Negative
Laurenzi2013	NA	Neutral	NA
Li2020	Positive	NA	NA
Mandel2013	Neutral	NA	NA
Rodríguez2019	Negative	NA	NA
Mahadin2022	NA	Negative	Negative
Turner2013	NA	Positive	NA
Miller2018	Positive	NA	Negative
Pandey2022	NA	Neutral	NA
Patil2014	Neutral	NA	NA
Pecina2014	NA	Negative	NA
Turner2014	Positive	NA	NA
Skianis2020	Positive	NA	NA
Taira2021	Neutral	NA	Negative
Turner2015a	Neutral	NA	Negative
Turner2015b	Neutral	NA	NA
Tensmeyer2022	NA	Positive	NA
Dharmawan2019	NA	Positive	NA
Guo2016	Neutral	Positive	NA
Takakusagi2021	Negative	Neutral	Negative
Taylor2015	Neutral	Neutral	NA

Way2020	Neutral	NA	NA
Wu2011	Neutral	Neutral	NA
Xie2021	Neutral	NA	NA
Yan2021	Neutral	NA	NA
Yepes2017	Neutral	NA	NA
Yang2023	Neutral	Positive	Neutral
Zeng-Treitler2010	Negative	Positive	Negative
Ziganshina2021	Neutral	NA	Neutral

The following abbreviations stand for 1. TR= “Technical Readiness”, 2.SEL= ˝Socioeconomic Readiness”, 3.ELR = “Ethicolegal Readiness”, 4.NA = “Not Addressed”.
